# Arboreal networks and their underlying trees

**DOI:** 10.1007/s00285-026-02364-8

**Published:** 2026-03-05

**Authors:** K. T. Huber, D. Overman

**Affiliations:** https://ror.org/029ss0s83 grid.440858.5 0000 0004 0381 4018 UEA, Norwich, England

**Keywords:** Arboreal network, Horizontal gene transfer, Enhanced quartet tree system, Augmented tree, Phylogenetic tree, Multiple-rooted network, 05C05, 05C20, 05C85, 05D15, 92D15

## Abstract

Horizontal gene transfer (HGT) is an important process in bacterial evolution. Current phylogeny-based approaches to capture it cannot however appropriately account for the fact that HGT can occur between bacteria living in different ecological niches. Due to the fact that arboreal networks are a type of multiple-rooted phylogenetic network that can be thought of as a forest of rooted phylogenetic trees along with a set of additional arcs each joining two different trees in the forest, understanding the combinatorial structure of such networks might therefore pave the way to extending current phylogeny-based HGT-inference methods in this direction. A central question in this context is, how can we construct an arboreal network? Answering this question is strongly informed by finding ways to *encode* an arboreal network, that is, breaking up the network into simpler combinatorial structures that, in a well defined sense uniquely determine the network. In the form of triplets, trinets and quarnets such encodings are known for certain types of single-rooted phylogenetic networks. By studying the underlying tree of an arboreal network, we complement them here with an answer for arboreal networks.

## Introduction

Evidence suggests that bacteria living in distinct ecological niches such as the human gut and the human skin can exchange genetic material via horizontal gene transfer (Jeong et al. 2019). Until now, the standard approach in phylogenetics to model this phenomenon has been to assume a common phylogenetic tree for the species of interest called a species tree and to then explain the evolutionary signal conveyed by a gene tree within the assumed species tree (Bansal et al. 2012; Jeong et al. 2019). To do this, a cost is generally assigned to permissible evolutionary events such as speciation, duplication, and loss and the task is then to find an embedding of the gene tree within the species tree that is optimal in terms of a cost function. Although much research has gone into extending and refining this approach by, for example, assuming additional evolutionary events such as incomplete lineage sorting, not much is known for the case where a species tree cannot be found with high enough confidence or where the information that the exchange of genetic material between niches is important and that this information should therefore be preserved.

**Fig. 1:**
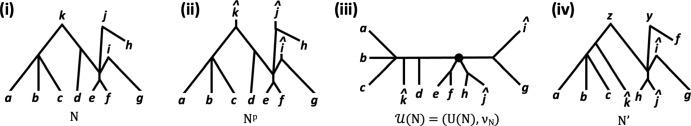
(i) An arboreal network *N* with leaf set $X=\{a,\ldots , h\}$ and root set $\{i,j,k\}$. (ii) The planted version $N^p$ of *N* with root set $\{\widehat{i},\widehat{j},\widehat{k}\}$. (iii) The augmented tree $\mathcal {U}(N)=(U(N),\nu _N)$ with leaf set $X\cup \{\widehat{i}, \widehat{j},\widehat{k}\}$ associated to *N* where $\nu _N$ is the map that assigns $\bullet$ to the indicated vertex and $\circ$ otherwise. For clarity purposes, only vertices that are assigned the value $\bullet$ under $\nu _N$ are indicated. (iv) With *z* and *y* denoting the vertices in (iii) adjacent with *d* and *e*, respectively, an arboreal network $N'$ on $\{a,b,c,\widehat{k},h,\widehat{j},f,g\}$ for which $\mathcal {U}(N')$ equals $\mathcal {U}(N)$ once we put $d=\widehat{z}$ and $e=\widehat{y}$

To start filling this gap, multiple-rooted phylogenetic networks such as the one depicted in Figure 1(i) have been introduced and studied in the literature in the form of Overlaid Species Forests (Huber et al. 2022a), forest-based networks (Huber et al. 2022b, 2025; Lafond and Moulton 2025) and arboreal networks (Huber et al. 2024a). Although distinct combinatorial objects all of whose set of leaves is a pre-given set *X* of organisms, they all can be thought of as a forest *S* of rooted phylogenetic trees (each representing, for example, the evolutionary past of a set of bacteria inhabiting an ecological niche) along with a set *A* of additional arcs (each representing, for example, a horizontal gene transfer event) such that each arc in *A* joins two distinct trees in *S*.

Arboreal networks *N* are at the centre of this paper and, in addition to being multiple-rooted, enjoy the property that their *underlying graph* i.e. the graph obtained from *N* by ignoring directions and suppressing all *roots* (i.e. vertices with indegree zero and outdegree two or more) that have become vertices of degree two are unrooted phylogenetic trees in the usual sense (see Sections 6 and 2 for formal definitions, respectively). Although relatively simple looking structures, arboreal networks enjoy interesting combinatorial properties such as being Ptolemaic (Huber et al. 2024a). Furthermore, they naturally generalize so called tree-based single-rooted phylogenetic networks (Francis and Steel 2015) which have attracted a considerable amount of attention in the literature (Fischer and Francis 2020; Francis et al. 2018; Hayamizu 2016; Suzuki et al. 2024; Zhang 2016). However in contrast to certain single-rooted phylogenetic networks not much is known about them in terms of encodings whereby we mean that the network can be broken up into simpler combinatorial structures that, in a well-defined sense, uniquely determine the network (see e.g. Gambette and Huber 2012; Huber and Moulton 2013; Van Iersel and Moulton 2014 for such encodings). Arguably, the main attraction of them is that they not only naturally lend themselves as a first port of call for developing reconstruction algorithms for arboreal networks so that their power can be exploited by evolutionary biologists, but also that they are generally easier to infer from real biological data.

As suggested by the example in Figure 1, any answer to the question of what can be said about encodings for arboreal networks *N* must in one way or another incorporate the roots of *N* and also its *reticulation vertices* that is, the vertices of indegree two or more and outdegree one. Motivated by the fact that a rooted phylogenetic tree is sometimes constructed from an unrooted phylogenetic tree by including an outgroup for rooting purposes, we address the former by associating to an arboreal network *N* with leaf set *X* its planted version $N^p$ by adding for each of the roots *r* of *N* a new vertex $\widehat{r}$ and an arc $(\widehat{r},r)$. Calling the vertices $\widehat{r}$ the *roots* of $N^p$ and denoting the set of newly added vertices by *R*(*N*) then it is immediately clear that the underlying graph $U(N^p)$ of $N^p$ is an unrooted phylogenetic tree with leaf set $X\cup R(N)$. Writing *U*(*N*) for $U(N^p)$ to keep notation at bay, we address the latter by recording the reticulation vertices of *N* in terms of a map $\nu _N$ from the vertex set of *U*(*N*) into a set $\{\bullet , \circ \}$ where a vertex is assigned $\bullet$ under $\nu _N$ if, in *N*, it is a reticulation vertex of *N* and $\circ$ otherwise. For the convenience of the reader, we depict for the arboreal network *N* in Figure 1(i) the planting $N^p$ of *N* and the augmented tree $\mathcal {U}(N)=(U(N),\nu _N)$ associated to *N* in Figures 1(ii) and (iii), respectively. Note that, from a conceptual level, such trees are unrooted phylogenetic trees *T* along with a symbolic map from the vertex set of *T* into a set of symbols which, in our case, are $\bullet$ and $\circ$ (see e.g. Böcker and Dress 1998; Hellmuth et al. 2013; Huber et al. 2019, 2024b and (Semple et al. (2003), Section 7.6) for other cases where such maps have been used in phylogenetics). Throughout the paper, we will refer to them as *augmented trees*.

As is well-known, unrooted phylogenetic trees are encoded by collections of unrooted phylogenetic trees on 4 leaves called quartet trees that satisfy certain properties (see e.g. Colonius and Schulze 1981; Dress and Erdös 2003; Grünewald et al. 2008; Semple et al. 2003). Using the novel concept of an *enhanced quartet tree system* which we introduce here, we present in Theorem 1 an encoding of augmented trees in terms of enhanced quartet tree systems that satisfy six non-trivial mutually independent properties. As the forbidden configuration for augmented trees in Figure 8 suggests, this characterization of augmented trees can however not easily be used as a starting point for finding a characterization of arboreal network in terms of such quartet tree systems. We therefore restrict our attention in the last-but-one section of the paper to binary augmented trees ${\mathcal {T}}$and make the additional assumption that the set of roots of an arboreal network *N* that is potentially obtainable from ${\mathcal {T}}$ is known. In particular, denoting the set of leaves of ${\mathcal {T}}$ by $R\cup X$ where we view *R* as the set of roots of *N* and *X* is the set of leaves of *N* then we present a $O(|X\cup R|^2)$ time algorithm that either constructs such an arboreal network from ${\mathcal {T}}$, *X* and *R* or returns the statement that no such arboreal network exists (see also Theorem 2).

The paper is organized as follows. In the next section, we present formal definitions of the concepts that allow us to establish Theorem 1. This also includes the definition of the enhanced quartet tree system $(\gamma _T,\nu _T)$ induced by an augmented tree ${\mathcal {T}}=(T,\nu )$. In Section 3, we introduce our aforementioned six properties for enhanced quartet tree systems and show that they are independent of each other. Subsequent to that section, we then establish some basic results about enhanced quartet tree systems which will allow us to prove Theorem 1 in Section 5. In Section 6, we then present our forbidden configuration and also the aforementioned algorithm, the run time of which we establish in Theorem 2.

## Preliminaries

From now on, $|X|\ge 4$. For a map $f:A\rightarrow B$ from a set *A* into a set *B* of integers, we define the *support* *supp*(*f*) of *f* to be the set $\{a\in A:\, f(a)\ge 0\}$.

Suppose for the following that *G* is a simple graph. Then we denote the vertex set of *G* by *V*(*G*) and the set of edges of *G* by *E*(*G*). Furthermore, we denote an edge between two distinct vertices *u* and *v* of *G* by $\{u,v\}$. We call a vertex of *G* of degree one a *leaf* of *G* and denote the set of leaves of *G* by *L*(*G*).

Suppose that $v\in V(G)$. Then we call *v* an *interior vertex* of *G* if *v* is not a leaf of *G*. If *u* is a further interior vertex of *G* such that $e=\{u,v\}\in E(G)$ then we call *e* an *interior edge* of *G*. If the degree of *v* is two then we call *v* a *subdivision point* of *G*. For a subdivision point *v* with adjacent vertices *w* and $w'$ we refer to the deletion of *v* and its two incident edges and addition of the edge $\{w,w'\}$ as *suppressing* of *v*. If $|V(G)|\ge 3$ and *x*, *y*, and *z* are three vertices of *G* such that *v* is the unique vertex that simultaneously lies on the path from *x* to *y*, on the path from *x* to *z* and on the path from *z* to *y*, then we call *v* the *median of* *x*, *y*, *and* *z*. In this case, we also write $med_G(x,y,z)$ for *v* where the order of *x*, *y* and *z* is of no relevance. Note that a graph might contain three vertices that do not have a median – see e. g.  Bandelt and Van De Vel (1999); Bruckmann et al. (2022); Choe et al. (2008); Hellmuth et al. (2023); Klavzar and Mulder (1999); Mulder (2011, 1978) for more on medians in graphs.

### Phylogenetic trees

Suppose that *T* is an unrooted tree with leaf set *X*. Then we say that *T* is *partially subdivided* if *T* only has finitely many subdivision points and there is no leaf that is adjacent with a subdivision point of *T*. If *T* does not have a subdivision point then *T* is called a *phylogenetic tree * (*on* *X*).

Suppose for the following that *T* is a phylogenetic tree on *X*. If *T* is such that every interior vertex of *T* has degree three, then we call *T* a *binary* phylogenetic tree and if *T* has a single interior vertex then we call *T* a *star tree*. We refer to a set *L* of leaves of *T* with at least two elements as a *multi-cherry* of *T* if there exists a unique vertex in *T* that is adjacent with every leaf in *L*. For a phylogenetic tree *T* on *X* and a non-empty subset $Y\subseteq X$ with at least four elements, we call the minimal connected subgraph of *T* that joins the elements in *Y* the *span* of *Y*, denoted by *T*(*Y*). Furthermore, we denote by $T|_Y$ the phylogenetic tree on *Y* obtained by suppressing all vertices of degree two in *T*(*Y*). We say that two phylogenetic trees *T* and $T'$ on *X* are *equivalent* if there exists a bijection $\psi :V(T)\rightarrow V(T')$ that gives rise to a graph isomorphism between *T* and $T'$ that is the identity on *X*. If *T* is binary and has four leaves then we call *T* a *quartet tree*. We denote the set of all quartet trees whose leaf set is contained in *X* by ${\mathcal {Q}}(X)$. We call a non-empty subset ${\mathcal {Q}}'\subseteq {\mathcal {Q}}(X)$ of quartets trees a *quartet tree system * (*on *$\bigcup _{q\in {\mathcal {Q}}'} L(q)$).

To be able to link a quartet tree system ${\mathcal {Q}}$ with the set $\mathcal {S}$ of partially subdivided quartet trees obtained from ${\mathcal {Q}}$ by adding $k_q\ge 0$ subdivision points to the interior edge of a quartet tree *q* in ${\mathcal {Q}}$, we next associate a map $\gamma :{\mathcal {Q}}(X)\rightarrow \mathbb {Z}_{\ge -1} = \{n \in \mathbb {Z}\,|\,n\ge -1\}$ to ${\mathcal {Q}}$ defined, for all $q\in {\mathcal {Q}}(X)$, as follows. If $q\not \in {\mathcal {Q}}$, then we put $\gamma (q)=-1$ and, otherwise, we put $\gamma (q)=k_q$. We call $\gamma$ a *subdivision map* for ${\mathcal {Q}}$ and also write ${\mathcal {Q}}_{\gamma }$ for $\mathcal {S}$. Furthermore, we call ${\mathcal {Q}}_{\gamma }$ a *system of partially subdivided quartet trees*. By abuse of terminology, we sometimes refer to the pair $(q,\gamma (q))$ with $q\in {\mathcal {Q}}$ as a partially subdivided quartet tree in ${\mathcal {Q}}_{\gamma }$ and to a partially subivided quartet tree $s\in {\mathcal {Q}}_{\gamma }$ with $k_s\ge 0$ subdivision points as a pair $(q_s,k_s)$ where $q_s$ is the quartet tree obtained from *s* by removing all its subdivision points.

Suppose for the following that *q* is a quartet tree in ${\mathcal {Q}}(X)$ with leaf set $\{a,b,c,d\}$. Let *s* and *t* denote the two interior vertices of *q*. If the path from *a* to *b* crosses *s* but not *t* (and therefore the path from *c* to *d* crosses *t* but not *s*) then we also denote *q* by *ab*|*cd* or, alternatively, by *cd*|*ab* where, in each case, the order of *a* and *b* and *c* and *d* does not matter. Suppose that *q* is a quartet tree in ${\mathcal {Q}}(X)$ and that *T* is a phylogenetic tree on *X*. Then we say that *q* is *displayed* by *T* if *q* is equivalent with $T|_{L(q)}$. We denote the set of all quartet trees displayed by *T* by ${\mathcal {Q}}(T)$.

**Fig. 2:**
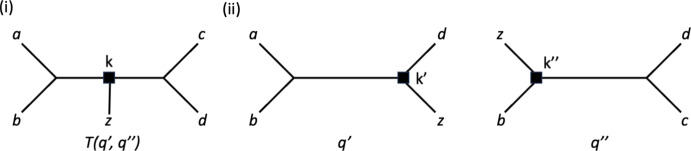
(i) The phylogenetic tree $T(q',q'')$ on $X:=\{a,b,c,d,z\}$ that simultaneously displays the two quartet trees $q':= ab|dz$ and $q'': = bz|dc$ in (ii). The vertices *k*, $k'$ and $k''$ are the medians of the vertices *b*, *z*, and *d* in the respective quartet trees and are indicated as squares

For $$X:=\{a,b,c,d,z\}$$ and two quartet trees $$q':= ab|dz$$ and $$q'':= zb|dc$$, we denote the unique binary phylogenetic tree on *X* that simultaneously displays $$q'$$ and $$q''$$ by $$T(q',q'')$$ – see Figure 2 for an illustration. Note that the five quartet trees that make up the system $${\mathcal {Q}}(T(q',q''))$$ of quartet trees displayed by $$T(q',q'')$$ can be obtained by taking, for example, the so-called semi-dyadic closure $$scl_2(q',q'') $$ of $$q'$$ and $$q''$$ – see Colonius and Schulze (1981) and Semple et al. (2003) for more on the semi-dyadic closure of a quartet tree system. The map $$\gamma :{\mathcal {Q}}(T(q',q''))\rightarrow \mathbb {Z}_{\ge -1}$$ that assigns the value 1 to the quartet tree *ab*|*cd*, the value 0 to all other quartet trees in $${\mathcal {Q}}(T(q',q''))$$, and the value $$-1$$ to the remaining quartet trees in $${\mathcal {Q}}(X)$$ is a subdivision map for $${\mathcal {Q}}(T(q',q'))$$.

### Phylogenetic trees and splits

A *partial split* $\sigma$ (*on X*) is a set $\{A,B\}$ where *A* and *B* are two non-empty disjoint subsets of *X*. If $A\cup B=X$, then we call $\sigma$ a *split* of *X*. In either case, we sometimes also write *A*|*B* (or, equivalently, *B*|*A*) for $\sigma$ and call *A* and *B* the *parts* of $\sigma$.

If one of the parts of a partial split $\sigma$ on *X* has size one then we call $\sigma$ a *trivial partial split* of *X*. For a subset $Y\subseteq X$ and a split $\sigma :=A|B$ on *X* such that $B':=Y\cap B\not =\emptyset$ and $A':=Y\cap A\not =\emptyset$, we call the split $A'|B'$ on *Y* the *restriction* of $\sigma$ to *Y*. We refer to a set $\varSigma$ of partial splits on *X* as a *system of partial splits* (*on X*), and as a *split system * (*on* *X*) if all elements of $\varSigma$ are splits on *X*. We say that a split $\sigma$ on *X* *displays* a partial split $\sigma '$ on *X* if there exists a subset $Y\subseteq X$ of *X* such that $\sigma '$ is the restriction of $\sigma$ to *Y*.

Clearly, deleting from a phylogenetic tree *T* on *X* an edge *e* induces a split $\sigma _e$ on *X* given by taking the leaf set of one of the resulting connected components to be one part of $\sigma _e$ and the leaf set of the other to be the other part of $\sigma _e$. We denote the split system obtained from *T* by taking all splits $\sigma _e$, $e\in E(T)$, by $\varSigma (T)$. We say that a split $\sigma$ is *displayed* by a phylogenetic tree *T* if $\sigma \in \varSigma (T)$. In case *T* is a quartet tree, then we call the split induced by deleting the interior edge of *T* the *quartet split* of *T*. We say that a split $\sigma$ of *X* *displays* a quartet tree $q\in {\mathcal {Q}}(X)$ if the quartet split associated to *q* is displayed by $\sigma$. Finally, for a non-trivial split $\sigma$ of *X*, we denote by ${\mathcal {Q}}(\sigma )$ the set of quartet trees in ${\mathcal {Q}}(X)$ that are displayed by $\sigma$.

Intriguingly, a phylogenetic tree can be reconstructed from its induced set of quartet trees in polynomial time. To make this more precise, we follow Semple et al. (2003) and denote for a quartet tree system ${\mathcal {Q}}$ on *X* the split system obtained by taking all splits *A*|*B* of *X* such that, for all pairwise distinct $a_1,a_2\in A$ and $b_1,b_2\in B$, we have $a_1a_2|b_1b_2\in {\mathcal {Q}}$ by $\varSigma ({\mathcal {Q}})$. By (Semple et al. (2003), Theorem 6.3.7), it follows for any phylogenetic tree *T* on *X* that the split system $\varSigma ({\mathcal {Q}}(T))$ associated to the set ${\mathcal {Q}}(T)$ of quartet trees displayed by *T* is the set of non-trivial splits displayed by *T*. Furthermore, $\varSigma ({\mathcal {Q}}(T))$ and therefore also *T* can be constructed from ${\mathcal {Q}}(T)$ in polynomial time - see (Dress et al. (2012), Chaptrer 8) for more on the relationship between quartet trees, quartet splits, and split systems.

### Augmented trees and enhanced quartet tree systems

Suppose for the following that *T* is a partially subdivided tree with leaf set *X*. Then we call a map $\nu :V(T)\rightarrow \{\circ , \bullet \}$ an *augmentation map* (*for* *T*) if, for all $v\in V(T)$, we have that $\nu (v)\in \{\circ , \bullet \}$ if *v* is an interior vertex of *T* that is not a subdivision point of *T*, $\nu (v)=\bullet$ if *v* is a subdivision point of *T*, and, otherwise, $\nu (v)=\circ$ holds.

Suppose that $\nu$ is an augmentation map for *T*. Then we call a subdivision point *v* of *T* an *augmentation point* and a non-subdivision point *v* of *T* with $\nu (v)=\bullet$ an *augmentation vertex* of *T* under $\nu$. We denote the set of augmentation vertices of *T* by $\mathcal {A}_{(T,\nu )}$, or $\mathcal {A}_T$, for short. We call a pair $\mathcal {T}=(T,\nu )$ an *augmented, partially subdivided tree* and refer to *T* as the *underlying tree of* $\mathcal {T}$. If *T* is a phylogenetic tree, then we refer to $\mathcal {T}$ as an *augmented (phylogenetic) tree* (*on* *X*). Note that a phylogenetic tree is an augmented tree without any augmentation vertices. Note also that in the context of reconstructing an arboreal network *N* from an augmented tree $(T, \nu )$ only limited information is provided by the augmentation map $\nu$ as to where to place the roots of *N* - see Figure 1 and also Section 6 for more on this.

Extending the notion of an augmentation map for a partially subdivided tree to a system $\mathcal {S}$ of partially subdivided quartet trees, we call a map $\nu : V(\mathcal {S}):=\bigcup _{q\in \mathcal {S}} V(q)\rightarrow \{\bullet ,\circ \}$ an *augmentation map for* $\mathcal {S}$ if, for all $v\in V(\mathcal {S})$, we have that $\nu (v)=\circ$ if *v* is a leaf of at least one partially subdivided quartet tree in $\mathcal {S}$, $\nu (v)=\bullet$ if *v* is a subdivision point of at least one such tree in $\mathcal {S}$, and, otherwise, $\nu (v)\in \{\bullet , \circ \}$. We sometimes refer to a set of augmented partially subdivided quartet trees as a *system of augmented, partially subdivided quartet trees*.

**Fig. 3:**
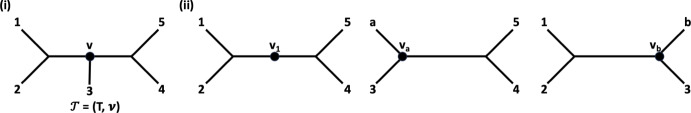
(i) An augmented tree ${\mathcal {T}}= (T,\nu )$ on $X=\{1,\ldots ,5\}$. For clarity purposes, only vertices that are assigned the value $\bullet$ under $\nu$ are indicated. (ii) With $a \in \{1,2\}$ and $b \in \{4,5\}$ the enhanced quartet tree system $(\gamma _T, \nu _T)$ induced by the augmented tree in (i). Again, only augmentation vertices and augmentation points under $\nu _T$ are indicate

To illustrate some of these concepts, consider the system $\mathcal {S}$ of augmented, partially subdivided quartet trees on $X:=\{1,\ldots ,5\}$ depicted in Figure 3. Ignoring the augmentations for the moment, then $\mathcal {S}$ comprises the partially subdivided quartet trees in part (ii) of that figure where $a \in \{1,2\}$ and $b \in \{4,5\}$. The augmentation map $\mu$ for $\mathcal {S}$ assigns the value $\bullet$ to the vertices $v_1$, $v_a$, and $v_b$ with $a\in \{1,2\}$ and $b\in \{4,5\}$ and the value $\circ$ to all other vertices in $V(\mathcal {S})$. Note that $v_1$ is an augmentation point of the partially subdivided quartet tree $q=12|34$ and that $v_2$ is an augmentation vertex of the quartet trees 13|45 and 23|45. Thus, $\mathcal {A}_{13|45}=\{v_1\}$.

We conclude this section with formalizing a central concept already mentioned in the introduction. We call a pair $(\gamma ,\nu )$ an *enhanced quartet tree system* (*on X*) if $\gamma :{\mathcal {Q}}(X)\rightarrow \mathbb {Z}_{\ge -1}$ is a subdivision map for the quartet trees in ${\mathcal {Q}}(X)$ such that $supp(\gamma )\not =\emptyset$ and $\nu :V(supp(\gamma )_{\gamma })\rightarrow \{\bullet ,\circ \}$ is an augmentation map for $supp(\gamma )_{\gamma }$.

As is straightforward to check, an augmented tree ${\mathcal {T}}$ on *X* whose underlying tree *T* is not a star tree gives rise to an enhanced quartet tree system $(\gamma _T, \nu _T)$ on *X* as follows.

- $\gamma _T: {\mathcal {Q}}(X) \rightarrow {\mathbb {Z}}_{\ge -1}$ is the map which maps each quartet tree $q\in {\mathcal {Q}}(X)$ to $-1$ if $q\not \in {\mathcal {Q}}(T)$ and if $q\in {\mathcal {Q}}(T)$ and *x*, *y*, *t*, *z* are distinct elements in *X* such that $q=xy|tz$ then $\gamma _T(q)$ is the number of augmentation vertices of *T* that are interior vertices on the path in *T* connecting the path from *x* to *y* and the path from *t* to *z*.
- $\nu _T:V({\mathcal {Q}}(T)_{{\gamma }_T})\rightarrow \{\circ ,\bullet \}$ is the map defined by putting $\nu _T(v)=\bullet$ if there exists a quartet tree in ${\mathcal {Q}}(T)$ such that, in ${\mathcal {T}}$, *v* is a vertex in $\mathcal {A}_{{\mathcal {T}}}$ and $\nu _T(v)=\circ$ otherwise.

Returning to the example of the augmented tree ${\mathcal {T}}=(T,\nu )$ on $X:= \{1,\ldots ,5\}$ depicted in Figure 3(i), we have that $\nu _T=\nu$ and that $\gamma _T$ is the map that assigns value "1" to the quartet tree 12|54, the value 0 to all other quartet trees in ${\mathcal {Q}}(T)$ and the value $-1$ to all other quartet trees in ${\mathcal {Q}}(X)$.

## Six useful properties and their independence

In this section, we introduce six useful properties called (A1)-(A6) for enhanced quartet tree systems. As we shall see in Section 5, they will allow us to characterize augmented trees in terms of such systems because the enhanced quartet tree system $(\gamma _T,\nu _T)$ induced by an augmented tree ${\mathcal {T}}=(T,\nu )$ satisfies them. To be able to state Condition (A4), we require further concepts which we introduce next.

Suppose that *T* is a phylogenetic tree on *X* and that $Y:=\{w, x,y,z\}$ is a subset of *X*. Let *q* be a quartet tree such that $L(q)=Y$. Then we say that the median $med_q(x,y,z)$ of *x*, *y*, and *z* in *q* *gives rise to* the median $med_T(x,y,z)$ of *x*, *y* and *z* in *T* if there is a subdivision $q_s$ of *q* such that $q_s$ is isomorphic with the span $T'$ of *Y* in *T* such that the underlying bijection $\chi : V(q_s)\rightarrow V(T')$ is the identity on *Y* and $\chi (med_{q_s}(x,y,z))= med_{T'}(x,y,z)$.

Suppose that $(\gamma ,\nu )$ is an enhanced quartet tree system on *X* and that $q=ab|cd$ is a quartet tree in $\mathcal {Q}(X)$ with $\gamma (q)\ge 1$. Let *s* be an augmentation point of the partially subdivided quartet tree $(q,\gamma (q))$ under $\nu$. Then we say that *s* is *supported* by $supp(\gamma )$ if there exist quartet trees $q':=bz|cd$ and $q'':= ab|dz$ in $supp(\gamma )$ such that *q* is displayed by $T:=T(q',q'')$ so that *s* gives rise to the median $k:=med_T(b,z,d)$ in *T*, the median $k':=med_{q'}(b,d,z)$ in $q'$ gives rise to *k* when restricting *T* to $\{b,c,d,z\}$, the median $k'':=med_{q''}(b,d,z)$ in $q''$ gives rise to *k* when restricting *T* to $\{a,b,d,z\}$, and $\nu (k') =\nu (k'') =\bullet$. In this case, we also say that $q'$ and $q''$ *support* *s*.

To illustrate these concepts, consider the enhanced quartet tree system $(\gamma ,\nu )$ where the subdivision map $\gamma :{\mathcal {Q}}(X) \rightarrow \mathbb {Z}_{\ge -1}$ is the map $\gamma _T$ for the phylogenetic tree $T:=T(q',q'')$ depicted in Figure 2 and $\nu :V(supp(\gamma )_{\gamma })\rightarrow \{\bullet ,\circ \}$ is the augmentation map given, for all $v\in V(supp(\gamma )_{\gamma })$ by putting $\nu (v)=\bullet$ if $v=med_q(b,z,d)$ where $q\in \{q',q'',ab|cz, az|dc\}$ or *v* is the sole degree 2 vertex in $supp(\gamma )_{\gamma }$ and, otherwise, $\nu (v)=\circ$. Calling that vertex *s*, then *s* is an augmentation point in the partially subdivided quartet tree (*ab*|*cd*, 1). That *s* is supported by $supp(\gamma )$ follows from the fact that the quartet tree *ab*|*cd* is displayed by *T* and *s* gives rise to the medians mentioned in the definition of when an augmentation point is supported by $supp(\gamma )$. Furthermore, $q'$ and $q''$ support *s*.

Suppose again that $(\gamma ,\nu )$ is an enhanced quartet tree system on *X*. Then, Properties (A1)-(A6) are as follows.

1. For all $a,b,c,d\in X$, at most one of the values $\gamma (ab|cd)$, $\gamma (ac|bd)$, and $\gamma (ad|bc)$ is non-negative.
2. For all $x\in X-\{a,b,c,d\}$, if $\gamma (ab|cd)\ge 0$ then
  $$\begin{aligned} \gamma (ab|cx)\ge 0 \text{ and } \gamma (ab|dx)\ge 0 \end{aligned}$$
  or
  $$\begin{aligned} \gamma (ax|cd)\ge 0 \text{ and } \gamma (bx|cd)\ge 0\text{. } \end{aligned}$$
3. If *q* and $q'$ are quartet trees in $supp(\gamma )$ that share three pairwise distinct leaves *x*, *y*, and *z*, then $\nu (med_q(x,y,z))=\nu (med_{q'}(x,y,z))$.
4. Every augmentation point of a partially subdivided quartet tree $(q,\gamma (q))$ with $q\in supp(\gamma )$ is supported by $supp(\gamma )$.
5. Suppose $a,b,c,d,e \in X$ are such that $\gamma (ab|cd)>\gamma (ab|ce)\ge 0$ holds. If $\nu (med_{ab|ce}(a,c,e))=\bullet$ then
  1 $$\begin{aligned} \gamma (ae|cd) = \gamma (ab|cd) - \gamma (ab|ce) - 1 \end{aligned}$$
  and, if $\nu (med_{ab|ce}(a,c,e))=\circ$ then
  2 $$\begin{aligned} \gamma (ae|cd) = \gamma (ab|cd) - \gamma (ab|ce) \end{aligned}$$
6. Suppose $a,b,c,d,e \in X$ are such that both $\gamma (ab|cd)\ge 0$ and $\gamma (bc|de) \ge 0$ hold. If $\nu (med_{ab|cd}(b,c,d)) = \bullet$ then
  3 $$\begin{aligned} \gamma (ab|de) = \gamma (ab|cd) + \gamma (bc|de) + 1 \end{aligned}$$
  and, if $\nu (med_{ab|cd}(b,c,d)) = \circ$ then
  4 $$\begin{aligned} \gamma (ab|de)=\gamma (ab|cd) + \gamma (bc|de) \end{aligned}$$

Before we start our investigation of Properties (A1)-(A6) with establishing independence relationships, we remark that if $(\gamma ,\nu )$ is an enhanced quartet tree system such that $\nu$ assigns the value $\circ$ to every vertex in $V(supp(\gamma )_{\gamma })$, then $\gamma$ is the all-zero map and (A3) and (A6) always hold. Furthermore, Properties (A4) and (A5) hold vacuously and Properties (A1) and (A2) are similar to the antisymmetry, symmetry and substitution properties used in (Bandelt and Dress (1986), Propositon 2) to characterize quaternary relations on *X* that are tree-like in the sense that there exists a potentially unresolved *X*-tree that induces that relation on *X*. In the context of this it should be noted that an *X*-tree is a generalization of a phylogenetic tree in that it is a tree *T* along with a labelling $\phi :X\rightarrow V(T)$ such that every vertex *v* of *T* of degree one or two must be contained in $\phi (X)$. On the other hand and although $\gamma$ is similar in spirit to an object called a quartet weight function introduced in Grünewald et al. (2008), it cannot be used to characterize edge-weighted phylogenetic trees as $\gamma$ does not satisfy (Grünewald et al. (2008), Theorem 1).

### The independence of each of the Properties (A1) - (A3) from the other five

To see that Property (A1) is independent of (A2) - (A6), consider the set $X:= \{a,b,c,d\}$ and the map $\gamma :{\mathcal {Q}}(X) \rightarrow \mathbb {Z}_{\ge -1}$ given by putting $\gamma (q) = 0$, for all $q\in {\mathcal {Q}}(X)$. Furthermore, let $\nu :V(supp(\gamma )_{\gamma })\rightarrow \{\bullet , \circ \}$ be the map given by $\nu (v)=\circ$, for all $v\in V(supp(\gamma )_{\gamma })$. Then it is straightforward to see that (A1) does not hold whereas all of (A2) - (A6) hold vacuously.

To see that Property (A2) is independent of (A1) and (A3) - (A6), consider the set $X:= \{a,b,c,d,e\}$ and the map $\gamma :{\mathcal {Q}}(X) \rightarrow \mathbb {Z}_{\ge -1}$ given by putting $\gamma (ab|cd) = 0$ and $\gamma (q)=-1$, for all other $q\in {\mathcal {Q}}(X)$. Furthermore, let $\nu :V(supp(\gamma )_{\gamma })\rightarrow \{\bullet , \circ \}$ be the map given by $\nu (v)=\circ$ if $v\in V(\{ab|cd\}_{\gamma })$. Then (A1) holds because $|supp(\gamma )| = 1$, and (A3) - (A6) hold vacuously.

To see that Property (A3) is independent of (A1), (A2), and (A4) - (A6), consider the set $X:= \{a,b,c,d,e\}$ and the map $\gamma :{\mathcal {Q}}(X) \rightarrow \mathbb {Z}_{\ge -1}$ given by putting $\gamma (ab|cd) = \gamma (ab|ce) = \gamma (ab|de) = 0$ and $\gamma (q)=-1$, for all other $q\in {\mathcal {Q}}(X)$. Furthermore, let $\nu :V(supp(\gamma )_{\gamma })\rightarrow \{\bullet , \circ \}$ be the map given by $\nu (med_{ab|ce}(a,b,c)) = \bullet$ and $\nu (v)=\circ$, for all other $v\in V(supp(\gamma )_{\gamma })$. Then it is again straightforward to check (A1) and (A2) hold. Furthermore, (A4) - (A6) hold vacuously.

### Property (A4) is independent of Properties (A1) - (A3), (A5), and (A6)

We start the discussion of the independence of Property (A4) from (A1) - (A3), (A5), and (A6), by remarking that if (A2), (A3) and (A6) hold then, using the notation in the definition of supporting an augmentation point, we can only have that (A4) does not hold if at least one of the quartet trees $q'$ and $q''$ is not contained in $supp(\gamma )$ or, if they are both contained in $supp(\gamma )$, that $med_{q'}(k')$ (and therefore also $med_{q''}(k'')$) is assigned $\circ$ under $\nu$.

To see the independence in the first case, consider the set $X:=\{a,b,c,d\}$, the map $\gamma :{\mathcal {Q}}(X)\rightarrow \mathbb {Z}_{\ge -1}$ given by $\gamma (ab|cd)=1$ and $\gamma (q)=-1$ for all other $q\in {\mathcal {Q}}(X)$, and the map $\nu :V(\{ab|cd\}_{\gamma })\rightarrow \{\bullet ,\circ \}$ given by $\nu (v) =\circ$ for all $v\in V(\{ab|cd\}_{\gamma })$. Then it is straightforward to see that Properties (A1) - (A3) and (A5) and (A6) all hold. However, (A4) does not hold because there exist no two quartet trees $q',q''\in supp(\gamma )$ such that *ab*|*cd* is displayed by $T(q',q'')$.

In the second case, consider the set $X = \{a,b,c,d,e\}$ and the map $\gamma :{\mathcal {Q}}(X) \rightarrow \mathbb {Z}_{\ge -1}$ given by putting $\gamma (ab|cd) =\gamma (ae|cd)=\gamma (be|cd)=1$, $\gamma (ab|ce)=\gamma (ab|de)=0$, and $\gamma (q) = -1$, for all other quartet trees $q\in {\mathcal {Q}}(X)$. Furthermore, let $\nu : V(supp(\gamma )_{\gamma }) \rightarrow \{\bullet ,\circ \}$ be the map given by putting $\nu (v) = \circ$, for all $v\in V(supp(\gamma )_{\gamma })$. Then it is straightforward to show that Properties (A1) - (A3) and (A5) and (A6) all hold. However, (A4) does not hold in view of the definition of the map $\nu$.

### Property (A5) is independent of Properties (A1) - (A4) and (A6)

To see that (A5) is independent of (A1) - (A4) and (A6), we distinguish for two quartet trees *ab*|*cd* and *ab*|*ce* in $supp(\gamma )$ for which $\gamma (ab|cd)>\gamma (ab|ce)\ge 0$ holds between the cases that $med_{ab|ce}(a,c,e)$ is assigned $\circ$ or $\bullet$ under $\nu$. We start with the case $\nu (med_{ab|ce}(a,c,e))=\bullet$.

**Fig. 4:**
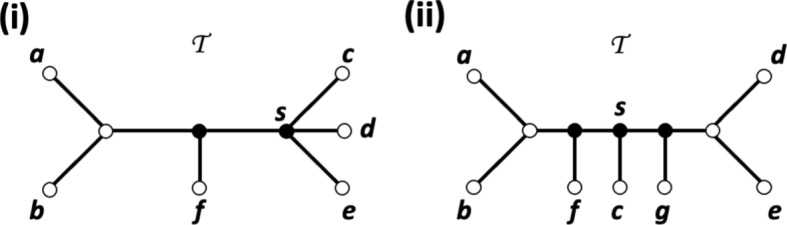
(i) The augmented tree ${\mathcal {T}}$ considered in the discussion of the case that Property (A5) is independent of Properties (A1) - (A4) and (A6) for the case of Equation (1). (ii) The augmented tree ${\mathcal {T}}$ considered in the discussion of the case that Property (A6) is independent of Properties (A1) - (A5) for the case of Equation (3)

Consider the set $X:= \{a,\ldots ,f\}$ and the map $\gamma :{\mathcal {Q}}(X)\rightarrow \mathbb {Z}_{\ge -1}$ whose support is given by the set of quartet trees displayed by the underlying tree *T* of the augmented tree ${\mathcal {T}}$ depicted in Figure 4(i). Put differently, $supp(\gamma )$ consists of the quartet trees *ab*|*xy* with $x,y\in \{c,d,e,f\}$ distinct and, for all $x,y\in \{e,c,d\}$ distinct, of the quartet trees of the form *ab*|*xy*, or *bf*|*xy* or *af*|*xy*. Put $\gamma (ab|cd) = 1$, $\gamma (q)= 0$ for all other quartet trees in ${\mathcal {Q}}(T)$, and $\gamma (q) = -1$ for all other $q\in {\mathcal {Q}}(X)$. Furthermore, let $\nu : V(supp(\gamma )_{\gamma } \rightarrow \{\bullet ,\circ \}$ be the indicated augmentation map. Put differently, $\nu (v)=\circ$ if *v* is a leaf of a quartet tree in $supp(\gamma )$ or $v= med_{ab|xy}(a,b,x)$ for some $x,y\in \{c,d,e,f\}$ distinct, and $\nu (v)=\bullet$ for all other $v\in V(supp(\gamma ))$.

The definition of $\nu$ combined with the fact that ${\mathcal {Q}}(T)=supp(\gamma )$ implies immediately that Properties (A1) - (A4) hold. To see that Property (A6) holds, it suffices to check the quartet tree pairs *q* and $q'$ where *q* is of the form *ab*|*fx* with $x\in \{c,d\}$ and $q'$ is of the form $q'=af|yz$ with $y,z\in \{c,d,e\}$ distinct. Since the vertex adjacent with *f* in a quartet tree in $supp(\gamma )$ is assigned the value $\bullet$ under $\nu$ and $\gamma (ab|cd)=1$, the definition of $\gamma$ on the remaining quartet trees in ${\mathcal {Q}}(T)$ implies that Equation (3) in (A6) holds. To see that Equation (1) in Property (A5) does not hold, note that $\gamma (ab|cd) =1>0= \gamma (ab|ce)$. Since $\nu (med_{ab|ce}(a,c,e))=\bullet$ Equation (1) implies that $-1=\gamma (ae|cd) = \gamma (ab|cd) - \gamma (ab|ce) - 1=0$ which is impossible.

To see that Property (A5) is independent of (A1) - (A4) and (A6) if $\nu (med_{ab|ce}(a,c,e))=\circ$ holds, consider the set $X:= \{a,b,c,d,e,f\}$ and the augmented tree ${\mathcal {T}}$ depicted in Figure 4(i) where we replace the augmentation of the vertex *s* in the augmentation map of ${\mathcal {T}}$ by $\circ$. Let $\gamma :{\mathcal {Q}}(X)\rightarrow \mathbb {Z}_{\ge -1}$ be defined as before, and let $\nu :V(supp(\gamma )_{\gamma })\rightarrow \{\bullet , \circ \}$ be the thus modified augmentation map. By definition, Properties (A1) - (A4) hold. Using the same arguments as before, it follows that Property (A6) also holds. To see that Equation (2) in Property (A5) does not hold, note again that $\gamma (ab|cd) > \gamma (ab|ce) \ge 0$. Since $\nu (med_{ab|ce}(a,c,e))=\circ$ Equation (2) implies that $-1=\gamma (ae|cd) = \gamma (ab|cd) - \gamma (ab|ce) =1$ which is impossible.

### Property (A6) is independent of Properties (A1) - (A5)

To see that Property (A6) is independent of (A1) - (A5), we distinguish for two quartet trees *ab*|*cd* and *bc*|*de* in $supp(\gamma )$ between the cases that $med_{ab|cd}(b,c,d)$ is assigned $\circ$ or $\bullet$ under $\gamma$. We start with the case $\nu (med_{ab|cd}(b,c,d))=\bullet$.

Consider the set $X:= \{a,\ldots ,g\}$ and the augmented tree ${\mathcal {T}}$ on *X* depicted in Figure 4(ii). Let *T* be the underlying tree of ${\mathcal {T}}$ and let $\gamma :{\mathcal {Q}}(X)\rightarrow \mathbb {Z}_{\ge -1}$ denote the map given by putting $\gamma (q)=1$ if $q\in {\mathcal {Q}}'=\{ab|de, ab|cd, ac|de, bc|de,$ $ab|ce\}$, $\gamma (q)=0$ if $q\in {\mathcal {Q}}(T)-{\mathcal {Q}}'$, and $\gamma (q)=-1$ if $q\in {\mathcal {Q}}(X)-{\mathcal {Q}}(T)$. Furthermore, let $\nu :V(supp(\gamma )_{\gamma })\rightarrow \{\bullet , \circ \}$ be the indicated augmentation map. Put differently, $\nu (v)=\circ$ if *v* is a leaf of a quartet tree in $supp(\gamma )$, or there exists a quartet tree of the from *ab*|*xy* with $x,y\in \{c,d,e,f,g\}$ distinct and $v=med_{ab|xy}(a,b,x)$, or there exists a quartet tree of the from *xy*|*de* with $x,y\in \{a,b,c,f,g\}$ distinct and $v=med_{xy|de}(d,e,x)$. Furthermore, $\nu (v)=\bullet$ for all other $v\in V(supp(\gamma )_{\gamma })$.

Clearly, Properties (A1) - (A3) hold. Also, it is straightforward to check that Properties (A4) and (A5) hold. Furthermore, $\nu (med_{ab|cd}(b,c,d))=\bullet$. However, Equation (3) in (A6) does not hold because $\gamma (ab|de)=1\not =3=\gamma (ab|cd)+\gamma (bc|de)+1$.

To see that (A6) is independent of (A1) - (A5) if $\nu (med_{ab|cd}(b,c,d))=\circ$, consider again the set $X:\{a,\ldots ,g\}$ and the augmented tree ${\mathcal {T}}$ on *X* depicted in Figure 4(ii) but with the augmentation of the vertex *s* replaced by $\circ$ in that augmentation map of ${\mathcal {T}}$. Let ${\mathcal {Q}}'$ and $\gamma :{\mathcal {Q}}(X)\rightarrow \mathbb {Z}_{\ge -1}$ be defined as before. Finally, let $\nu : (\gamma )_{\gamma })to \{\bullet ,\circ \}$ be the thus modified augmentation map. Then, as before, (A1) - (A5) hold. Furthermore, $\nu (med_{ab|cd}(b,c,d))=\circ$. However, Equation (4) in (A6) does not hold because $\gamma (ab|de)=1\not =2=\gamma (ab|cd)+\gamma (bc|de)$.

## Basic properties of enhanced quartet tree systems

In this section, we investigate properties of enhanced quartet tree systems. We start by showing that an augmented tree that is not a star tree gives rise to an enhanced quartet tree system that satisfies Properties (A1) - (A6). To be able to relate enhanced quartet tree systems with splits which we do next, we start with the following result whose Part (ii) is adapted from (Grünewald et al. (2008), Lemma 1).

### Lemma 1

Suppose that $(\gamma ,\nu )$ is an enhanced quartet tree system on *X* that satisfies Properties (A1) and (A2).

(i) If $a,b,c,d,e\in X$ are such that $\gamma (ab|ce), \gamma (ab|de) \ge 0$, then $\gamma (ab|cd) \ge 0$.
(ii) If $(\gamma ,\nu )$ also satisfies Properties (A3), (A5), and (A6), then the following property also holds:
  (A7) For all $$a,b,c,d,e \in X$$, $$\gamma (ab|cd)\ge \min \{\gamma (ab|ce),\gamma (ab|de)\}$$.

### Proof

(i) Assume for contradiction that $\gamma (ab|cd) = -1$. Applying (A2) to *ab*|*ce* and *d* implies that both $\gamma (ab|cd) \ge 0$ and $\gamma (ab|de) \ge 0$ or that $\gamma (ad|ce)\ge 0$ and $\gamma (bd|ce)\ge 0$. Since, by assumption, $\gamma (ab|cd) = -1$, it follows that $\gamma (ad|ce) \ge 0$. Similarly, applying (A2) to $\gamma (ab|de)$ and *c* implies that $\gamma (ac|de) \ge 0$. But this is impossible in view of (A1).

(ii) Suppose that Properties (A3), (A5) and (A6) also hold but that (A7) does not hold, that is,

5 $$\begin{aligned} \gamma (ab|cd) < \min \{\gamma (ab|ce),\gamma (ab|de)\} \end{aligned}$$

We claim first that

$$\begin{aligned} \gamma (ab|cd) \ge 0 \end{aligned}$$

To see the claim, assume that $\gamma (ab|cd) = -1$. Then Inequality (5) implies that $\gamma (ab|ce) \ge 0$ and $\gamma (ab|de) \ge 0$. Applying (A2) to $\gamma (ab|ce) \ge 0$ and *d* and noting that $\gamma (ab|cd) = -1$, we obtain $\gamma (bd|ce) \ge 0$. Similarly, applying (A2) to $\gamma (ab|de) \ge 0$ and *c*, yields $\gamma (bc|de) \ge 0$, a contradiction to Property (A1). Hence, $\gamma (ab|cd) \ge 0$ as claimed. By Inequality (5), $\gamma (ab|de)> 0$.

Using Inequality (5), we obtain $\gamma (ab|ce) > \gamma (ab|cd) \ge 0$. Note that, by Property (A3), $\nu (med_{ab|cd}(a,c,d))= \nu (med_{ad|ce}(a,c,d))$. Hence, by (A5), we have $\gamma (ad|ce) = \gamma (ab|ce) - \gamma (ab|cd)$ if $\nu (med_{ab|cd}(a,c,d)) = \circ$ and $\gamma (ad|ce) = \gamma (ab|ce) - \gamma (ab|cd) - 1$, otherwise. Thus, $\gamma (ad|ce)\ge 0$. Since $\gamma (ab|cd) \ge 0$ and therefore $\gamma (ad|bc) = -1$ because of Property (A1), we obtain $\gamma (bd|ce) \ge 0$ by applying (A2) to $\gamma (ad|ce)$ and *b*. In view of (A3), $\nu (med_{ab|cd}(b,c,d))= \nu (med_{bd|ce}(b,c,d))$ and so, by (A6),

$$ \gamma (ab|ce)=\gamma (ab|cd)+\gamma (bd|ce) $$

holds if $\nu (med_{ab|cd}(b,c,d)) = \circ$. Combined with Property (A3), it follows that $\nu (med_{bd|ce}(b,d,e))=\nu (med_{ab|de}(b,d,e))=\nu (med_{ab|cd}(b,c,d))$ since the phylogenetic tree $T'=T(bd|ce, ab|de)$ also displays the quartet tree *ab*|*cd* and $med_{T'}(b,d,e)=med_{T'}(b,c,d)$. By Property (A6),

$$ \gamma (ab|ce)=\gamma (ab|de)+\gamma (bd|ce) $$

must hold too in this case. Similar arguments imply that $\gamma (ab|de)+\gamma (bd|ce)+1=\gamma (ab|ce)=\gamma (ab|cd)+\gamma (bd|ce)+1$ holds in case $\nu (med_{ab|cd}(b,c,d)) = \bullet$. In either case, $\gamma (ab|de) = \gamma (ab|cd)$ follows, contradicting Inequality (5). $\square$

### Lemma 2

Suppose $(\gamma ,\nu )$ is an enhanced quartet tree system that satisfies Properties (A1)-(A3), (A5), and (A6). If $ss'|tt'$ is a quartet tree in $supp(\gamma )$ and *A*, *B* are disjoint subsets of *X* such that $s, s' \in A$, $t,t' \in B$, ${\mathcal {Q}}(A|B)\subseteq supp(\gamma )$, and $|A|+|B|$ is maximum, then *A*|*B* is a split of *X*.

### Proof

Suppose that the elements *s*, $s'$, *t* and $t'$ and the sets *A* and *B* are as in the statement of the lemma and assume for contradiction that *A*|*B* is not a split of *X*. Then there is an element $x\in X-(A\cup B)$. We claim that there exist (not necessarily distinct) elements $a_1,a_2,a_3\in A$ and $b_1,b_2,b_3\in B$ with $|\{a_1,a_2,b_1,b_2\}|=4$ such that

$$\begin{aligned} \gamma (a_1a_2|b_3x) = -1 \text{ and } \gamma (a_3x|b_1b_2) = -1\text{. } \end{aligned}$$

Indeed, if no such elements $a_1,a_2,a_3\in A$ and $b_1,b_2,b_3\in B$ existed then, since $A\cap B=\emptyset$ we have that there exists some $C\in \{A,B\}$ and some $D\in \{A,B\}-\{C\}$ such that $C\cup \{x\}$ and *D* are disjoint subsets of *X*. Without loss of generality, we may assume that $C=A$, that is, $A'=A\cup \{x\}$ and *B* are disjoint subsets of *X*. Since, by assumption, $\gamma (aa'|bb') \ge 0$ for all $a,a'\in A$ and $b,b'\in B$, it follows that $\gamma (aa'|bb') \ge 0$ for all $a,a'\in A'$ and $b,b'\in B$. Since $ss'|tt'\in Q(A'|B)$ and $|A|+ |B| <|A'|+|B|$ this is impossible as $|A|+|B|$ is maximum. Thus, the claim holds.

Since, by (A7), $\gamma (a_1a_2|b_3x) \ge \min \{\gamma (a_1a_2|b_3b),\gamma (a_1a_2|bx)\}$ for all $b \in B - \{b_3\}$ and $\gamma (a_1a_2|b_3x)=-1$, it follows for all such *b* that $\gamma (a_1a_2|bx) = -1$ because $a_1a_2|b_3b\in Q(A|B)\subseteq supp(\gamma )$. Similarly, $\gamma (ax|b_1b_2) = -1$ for all $a \in A-\{a_3\}$. Putting $a= a_1$ and $b = b_1$, we obtain $\gamma (a_1a_2|b_1x) =- 1$ and $\gamma (a_1x|b_1b_2) =-1$. But this is impossible since $\gamma (a_1a_2|b_1b_2) \ge 0$ and (A2) holds for $\gamma (a_1a_2|b_1b_2)$ and *x*. Hence, *A*|*B* is a split of *X*. $\square$

To be able to prove our next result, we require further notation. Suppose that $(\gamma ,\nu )$ is an enhanced quartet tree system and that $\sigma$ is a split contained in $\varSigma (supp(\gamma ))$. Then we denote by ${\mathcal {Q}}(\sigma )^-\subseteq {\mathcal {Q}}(\sigma )$ the set of all quartet trees $q\in {\mathcal {Q}}(\sigma )$ such that $\sigma$ is the sole split in $\varSigma (supp(\gamma ))$ that displays *q*. To help illustrate this definition, we return to the enhanced quartet tree system $(\gamma ,\nu )$ that we used to illustrate the concept of an augmentation point being supported by $supp(\gamma )$. Then since $\sigma =\{a,b\}|\{c,d,z\}$ is the sole split that displays the quartet tree *ab*|*dz*, we have $ab|dz \in {\mathcal {Q}}(\sigma )^-$. However $ab|cd\not \in {\mathcal {Q}}(\sigma )^-$ because *ab*|*cd* is also displayed by the split $\{a,b,z\}|\{c,d\}$.

### Lemma 3

Suppose $(\gamma ,\nu )$ is an enhanced quartet tree system on *X* that satisfies Properties (A1) - (A6).

If $\sigma$ is a split of *X* such that ${\mathcal {Q}}(\sigma )\subseteq supp(\gamma )$, then $\gamma (q)=0$, for all $q\in {\mathcal {Q}}(\sigma )^-$.

### Proof

Suppose that $\sigma =A|B$ is a split of *X* such that ${\mathcal {Q}}(\sigma )\subseteq supp(\gamma )$. Assume for contradiction that there exists some quartet tree $q=ab|cd\in {\mathcal {Q}}(\sigma )^-$ such that $\gamma (q)\ge 1$. Then the partially subdivided quartet tree $(q,\gamma (q))$ has at least one augmentation point *s*. Without loss of generality, we may assume that *s* is such that there is no further augmentation point of $(q,\gamma (q))$ between *s* and $med_q(b,c,d)$. By Property (A4), *s* is supported by $supp(\gamma )$. Hence, there exists some $z\in X-\{a,b,c,d\}$ and quartet trees $q' = ab|dz$ and $q''=bz|cd$ in $supp(\gamma )$ such that *q* is displayed by $T:=T(q',q'')$, the median $med_T(b,z,d)$ in *T* gives rise to the median $s'=med_{q'}(b,d,z)$ in $q'$ when restricting *T* to $\{a,b,d,z\}$ and the median $s''=med_{q''}(b,d,z)$ in $q''$ when restricting *T* to $\{b,c,d,z\}$, and $\nu (s')=\nu (s'')=\bullet$.

Let $A'$ and $B'$ be disjoint subsets of *X* such that $a,b\in A'$ and $d,z\in B'$, ${\mathcal {Q}}(A'|B')\subseteq supp(\gamma )$ and $|A'|+|B'|$ is maximum. Furthermore, let $A''$ and $B''$ be disjoint subsets of *X* such that $b,z\in A''$ and $c,d\in B''$, ${\mathcal {Q}}(A''|B'')\subseteq supp(\gamma )$ and $|A''|+|B''|$ is maximum. Note that such sets $A'$ and $B'$ must exist because $q'\in supp(\gamma )$ and that $A''$ and $B''$ must exist because $q''\in supp(\gamma )$. Since $(\gamma ,\nu )$ satisfies Properties (A1) - (A6), it follows by Lemma 2 that $A'|B'$ and $A''|B''$ are splits of *X*. Since, by construction, $q\in {\mathcal {Q}}(A'|B')$ and $q\in {\mathcal {Q}}(A''|B'')$ and either $z\in A$ or $z\in B$ must hold because $\sigma$ is a split of *X*, at least one of $A'|B'$ and $A''|B''$ cannot be $\sigma$, a contradiction because $q\in {\mathcal {Q}}(\sigma )^-$. $\square$

## Characterizing augmented trees

One of the fundamental results in phylogenetics is the Split-Equivalence Theorem (Semple et al. (2003), Theorem 3.1.4) – see also Buneman (1971) which may be stated as follows. Saying that a split system $\varSigma$ is *compatible* if for any two distinct splits $\sigma$ and $\sigma '$ in $\varSigma$ there exists a part $A\in \sigma$ and a part $A'\in \sigma '$ such that $A\cap A'=\emptyset$ then that theorem guarantees that for any split system $\varSigma$ on *X* that contains all trivial splits on *X*, there is a phylogenetic tree *T* on *X* with $\varSigma =\varSigma (T)$ if and only if $\varSigma$ is compatible. Moreover, if such a phylogenetic tree *T* exists, then, up to equivalence, *T* is unique. Our first result (Theorem 1) may be viewed as its analogue with the enhanced quartet tree system $(\gamma _T,\nu _T)$ induced by an augmented tree ${\mathcal {T}}=(T,\nu )$ introduced at the end of Section 2.3 playing a key role. To be able to state it, we require further concepts. We say that two augmented trees $(T,\nu )$ and $(T',\nu ')$ are *augmentation-equivalent* if *T* and $T'$ are equivalent as phylogenetic trees and, with $\psi :V(T)\rightarrow V(T')$ denoting the underlying bijection, we have for all $v\in V(T)$ that $\nu (v)=\nu '(\psi (v))$.

### Theorem 1

Suppose that $|X|\ge 4$ and that $(\gamma ,\nu )$ is an enhanced quartet tree system on *X*. Then there exists an augmented tree ${\mathcal {T}}=(T,\nu )$ such that *T* is not the star tree, $\gamma =\gamma _T$ and $\nu =\nu _T$ if and only if $(\gamma ,\nu )$ is an enhanced quartet tree system on *X* that satisfies Properties (A1)-(A6). Moreover, if such an augmented tree ${\mathcal {T}}$ exist then, up to augmentation-equivalence, ${\mathcal {T}}$ is unique.

### Proof

First suppose that ${\mathcal {T}}$ is an augmented tree on *X* such that its underlying tree of *T* is not a star tree. Then, the pair $(\gamma _T,\nu _T)$ introduced in Section 2.3 is an enhanced quartet tree system on *X*. We need to show that $(\gamma _T,\nu _T)$ satisfies Properties (A1) - (A6).

To improve the clarity of our arguments, we first assume that $|X|=4$. Then *T* is a quartet tree and $\gamma _T(T)=0$ since an augmented tree cannot contain augmentation points. Since $supp(\gamma _T)\subseteq \mathcal {Q}(T)=\{T\}$ it follows that *T* is the sole element in $supp(\gamma _T)$. Thus Property (A1) must hold. Since the remaining five properties require *X* to have at least five elements, it follows that they hold vacuously.

Assume for the remainder of this direction of the proof that $|X|\ge 5$. To see that $(\gamma _T,\nu _T)$ satisfies Property (A1), suppose that *a*, *b*, *c*, and *d* are pairwise distinct elements in *X*. Since a phylogenetic tree on *X* can display at most one quartet tree on $Y=\{a,b,c,d\}$, it follows that at most one quartet tree on *Y* can be assigned a non-negative value under $\gamma _T$. Thus, $(\gamma _T,\nu _T)$ satisfies (A1).

To see that $(\gamma _T,\nu _T)$ satisfies Property (A2), suppose that $a,b,c,d\in X$ are such that *ab*|*cd* is a quartet tree in $supp(\gamma _T)$. Then $\varSigma (T)$ contains a split $\sigma =A|B$ that displays *ab*|*cd*. Without loss of generality, we may assume that $a,b\in A$ and $c,d\in B$. Let $x\in X-\{a,b,c,d\}$. Then either $x\in A$ or $x\in B$. If $x\in A$, then $\sigma$ displays the quartet trees *ax*|*cd* and *bx*|*cd*. Hence, $ax|cd\in supp(\gamma _T)$ and $bx|cd\in supp(\gamma _T)$. Since similar arguments apply if $x\in B$, it follows that $(\gamma _T,\nu _T)$ satisfies (A2).

To see that $(\gamma _T,\nu _T)$ satisfies Property (A3), suppose that *q* and $q'$ are quartet trees in $supp(\gamma _T)$ that share three pairwise distinct leaves *x*, *y*, and *z*. Then the medians $med_q(x,y,z)$ and $med_{q'}(x,y,z)$ in *q* and $q'$, respectively, result from the median $med_T(x,y,z)$ in *T* when restricting *T* to the leaf sets of *q* and $q'$, respectively. Hence, $\nu _T(med_q(x,y,z))=\nu _T(med_{q'}(x,y,z))$. Thus, $(\gamma _T,\nu _T)$ satisfies (A3).

To see that $(\gamma _T,\nu _T)$ satisfies Property (A4), suppose that *s* is an augmentation point of a partially subdivided quartet tree $(q,\gamma (q))$ where $q:=cd|az$ is a quartet tree in $supp(\gamma _T)$. Then *s* must be an augmentation vertex of *T*. Since an augmentation point of a partially subdivided quartet tree can, in *T*, only be adjacent to at most one leaf of *T* and $|X|\ge 5$, there must exist interior vertices *v* and $v'$ of *T* such that *v* lies on the path joining *s* and *c* and $v'$ lies on the path joining *s* and *a*. Thus, $e:=\{s,v\}$ and $e':=\{s,v'\}$ are interior edges of *T* that lie on the path joining *c* and *a*. Choose a leaf *b* of *T* such that the path from *s* to *b* neither crosses *v* nor $v'$. Let $\sigma _e$ and $\sigma _{e'}$ denote the splits in $\varSigma (T)$ obtained by deleting the edges *e* and $e'$, respectively. Then since $q':=cd|bz$ is displayed by $\sigma _e$ and $q'':=bc|az$ by $\sigma _{e'}$, it follows that $q'$ and $q''$ are contained in $\mathcal {Q}(T)$. Thus, $q'$ and $q''$ are contained in $\mathcal {Q}(X)$ and $\gamma _T(q'),\gamma _T(q'')\ge 0$ holds. Since the restriction $T|_Y$ of *T* to $Y:=\{a,b,c,d,z\}$ is equivalent with the phylogenetic tree $T(q',q'')$, it follows that the median $med_T(b,c,z)$ in *T* results in the median $s'=med_{q'}(b,c,z)$ in $q'$ when restricting *T* to $\{b,c,d,z\}$ and the median $s''=med_{q''}(b,c,z)$ in $q''$ when restricting *T* to $\{a,b,c,z\}$. Since *s* is an augmentation vertex of *T*, it follows that $\nu _T(s') =\nu (s'') =\bullet$. Hence, *s* is supported by $supp(\gamma _T)$. Thus, $(\gamma _T,\nu _T)$ satisfies (A4).

To show that $(\gamma _T,\nu _T)$ satisfies Property (A5), suppose $a,b,c,d,e\in X$ are such that the quartet trees *ab*|*cd* and $q:=ab|ce$ are both contained in $supp(\gamma _T)$ and that $\gamma _T(ab|cd)>\gamma _T(q)\ge 0$. Let $q':=ae|cd$. Then the restriction $T|_Y$ of *T* to $Y:=\{ a,b,c,d,e\}$ must be equivalent with $T(q, q')$ since otherwise $ab|ce\not \in \mathcal {Q}(T)$ or $\gamma _T(ab|cd)\le \gamma _T(q)$ would hold which is impossible. It follows that the median $v:=med_q(a,c,e)$ in *q* results from the median $med_{T(q,q')}(a,c,e)$ in $T(q,q')$ which itself results from the median $w=med_T(a,c,e)$ in *T*. Similarly, the median $v':=med_{q'}(a,c,e)$ in $q'$ results from *w* this way. Thus, if *w* is not an augmentation vertex of *T* then $\nu _T(v)=\nu _T(v')=\circ$ and if *w* is an augmentation vertex of *T* then $\nu _T(v)=\nu _T(v')=\bullet$. Since *w* is an interior vertex on the path in *T* that joins the path from *a* to *b* in *T* and the path from *c* to *d* in *T*, Equation (1) follows if $\nu _T(v)=\bullet$. And if $\nu _T(v)=\circ$ then Equation (2) follows. Thus, $(\gamma _T,\nu _T)$ satisfies (A5).

Finally, to see that $(\gamma _T,\nu _T)$ satisfies Property (A6), suppose that there exist elements $a,b,c,d,e\in X$ such that the quartet trees $q:=ab|cd$ and $q':=bc|de$ are both contained in $supp(\gamma _T)$. Then there exists splits $\sigma :=A|B$ and $\sigma '\in \varSigma (T)$ such that $\sigma$ displays *q* and $\sigma '$ displays $q'$. Without loss of generality, we may assume that $a,b\in A$ and $c,d\in B$. Note that $e\not \in A$ because otherwise $q'\in supp(\gamma _T)$ would not hold. Hence, $e\in B$. In particular, the number of augmentation vertices in the interior of the path in *T* separating the path from *a* to *b* from the path from *e* to *d* equals $\gamma _T(q)+\gamma _T(q')+1$ if $w:=med_T(b,c,d)\in \mathcal {A}_T$ as in this case $\nu _T(med_{q}(b,c,d))=\bullet =\nu _T(med_{q'}(b,c,d))$. If $w \not \in \mathcal {A}_T$ then $\gamma _T(q)+\gamma _T(q')$ follows because in this case $\nu _T(med_{q}(b,c,d))=\circ =\nu _T(med_{q'}(b,c,d))$. Hence $(\gamma _T,\nu _T)$ satisfies (A6).

In summary, it follows that $(\gamma _T,\nu _T)$ is an enhanced quartet tree system on *X* that satisfies Properties (A1) - (A6).

To prove the converse, suppose that $(\gamma ,\nu )$ is an enhanced quartet tree system on *X* that satisfies Properties (A1) - (A6). We perform induction on $|supp(\gamma )|$. Since $supp(\gamma )\not =\emptyset$ by the definition of an enhanced quartet tree system, the base case is $|supp(\gamma )|=1$. Let *q* denote the sole quartet tree in $supp(\gamma )$. Then $\gamma (q)=0$ must hold since otherwise $(q,\gamma (q))$ contains an augmentation vertex which, in view of Property (A4) holding, must be supported by $supp(\gamma )$. But then $|supp(\gamma )|\ge 3$ which is impossible as we are in the base case of the induction. Thus, $(q,\nu )$ is an augmented tree on *X* for which $\gamma =\gamma _q$, and $\nu =\nu _q$ holds. Thus, the base case holds.

Assume for the remainder that $|supp(\gamma )|\ge 2$ and that the induction hypothesis holds for all enhanced quartet tree systems $(\gamma ',\nu ')$ on *X* with $|supp(\gamma ')|<|supp(\gamma )|$. Let $p:=a_1a_2|b_1b_2$ be a quartet tree in $supp(\gamma )$ such that $\gamma (p)$ is minimum. In view of (A4), it follows that $\gamma (p)=0$. Let *A* and *B* be two disjoint subsets of *X* such that $a_1,a_2\in A$, $b_1,b_2\in B$, ${\mathcal {Q}}(A|B)\subseteq supp(\gamma )$ and $|A|+|B|$ is maximum. Note that such subsets must exist since $p\in supp(\gamma )$. By Lemma 2, $\sigma :=A|B$ is a split of *X*. Choose a quartet tree $q^*:=ss'|tt'$ in ${\mathcal {Q}}(\sigma )^-$. Note that such a quartet tree must exist if $|\varSigma (supp(\gamma ))|=1$ since ${\mathcal {Q}}(\sigma )^-={\mathcal {Q}}(\sigma )$ holds in this case. If $|\varSigma (supp(\gamma ))|\ge 2$ then its existence follows from the fact that $\varSigma (supp(\gamma ))$ is compatible in view of Property (A1) holding. Indeed since $\varSigma (supp(\gamma ))$ is compatible, the Splits-Equivalence-Theorem reviewed at the beginning of this section, implies that there exists a phylogenetic tree *T* on *X* such that $\varSigma (T)= \varSigma (supp(\gamma ))$. Hence, there exists an interior edge *e* of *T* such that when deleting *e* one of the resulting connected components contains *A* in its vertex set and the other contains *B*. Let *v* and *u* denote the interior vertices of *T* incident with *e*. Let $l_v$ and $l_v'$ denote leaves of *T* such that the path joining $l_v$ and $l_v'$ crosses *v* but not *e*. Similarly, let $l_u$ and $l_u'$ denote leaves of *T* such that the path joining $l_u$ and $l_u'$ crosses *u* but not *e*. Then, by constructiion, the quartet tree $l_vl_v'|l_ul_u'$ is contained in $Q(\sigma )^-$ and we can take that tree to be $q^*$. Since ${\mathcal {Q}}(\sigma )\subseteq supp(\gamma )$ it follows by Lemma 3 that $\gamma (q)=0$ holds for all $q\in {\mathcal {Q}}(\sigma )^-$.

If ${\mathcal {Q}}(\sigma )^-=supp(\gamma )$ then consider the augmented tree ${\mathcal {T}}=(T,\mu )$ on *X* such that $\varSigma (T)$ comprises $\sigma$ and all trivial splits of *X* and $\mu : V(T)\rightarrow \{\circ ,\bullet \}$ is the map given by putting $\mu (x)=\nu (x)$ for all $x\in X$, $\mu (med_T(s,s',t))=\nu (med_{q^*}(s,s',t))$, and $\mu (med_T(s,t',t))=\mu (med_{q^*}(s,t',t))$. Note that *T* has a single interior edge and that *A* and *B* are multi-cherries of *T*. Also note that, by (A3), $\mu$ is well-defined. By construction, $\gamma _T=\gamma$ and $\mu =\nu _T$. Thus, this direction of the theorem holds in this case.

**Fig. 5:**
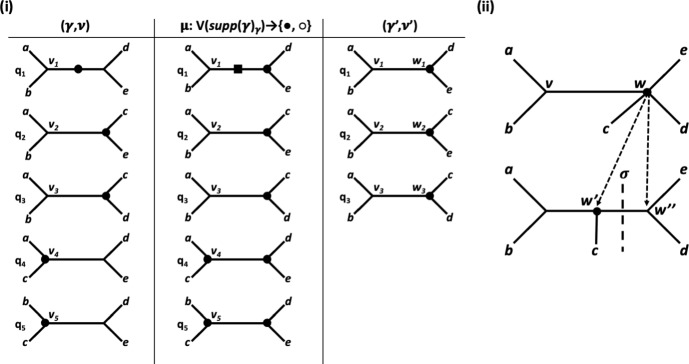
An example to illustrate the definitions of the maps $\mu$, $\nu '$ and $\gamma '$ used in the inductive part of the proof of Theorem 1. (i) For $X:=\{a,b,c,d,e\}$, the table gives the original enhanced quartet $(\gamma ,\nu )$ (left), the map $\mu$ (middle), and the enhanced quartet tree system $(\gamma ',\nu ')$ (right). The subdivision point of $q_1$ indicated in terms of a square is assigned the vaule $\circ$ under $\mu$. (ii) The augmented tree ${\mathcal {T}}'$ on *X* with underlying tree $T'$ and $\gamma _{T'}=\gamma '$ and $\nu _{T'}=\nu '$ holding is obtained using the induction hypothesis (top). The augmented tree ${\mathcal {T}}$ on *X* with underlying tree *T* and $\gamma _T=\gamma$ and $\nu _T=\nu$ holding is obtained by replacing the vertex *w* of $T'$ by the edge $\{w',w''\}$ whose deletion results in the split $\sigma$. In (i) and (ii), only vertices that have been assigned the value $\bullet$ by the map in question are indicated. All other vertices are assigned the value $\circ$ by that map. The vertices $v_1,\ldots , v_5$ correspond to the vertex *v* in (ii) and the vertices $w_1,w_2,w_3$ and the degree 2 vertex in $q_1$ correspond to the vertex *w*

So assume that ${\mathcal {Q}}(\sigma )^-\not =supp(\gamma )$. We next associate an enhanced quartet tree system $(\gamma ',\nu ')$ on *X* to $(\gamma ,\nu )$ and show that $(\gamma ',\nu ')$ satisfies the induction hypothesis. To help convey the main idea underpinning the construction of $(\gamma ',\nu ')$, we refer the interested reader to the example in Figure 5 which we also explain in more detail after the definition of $(\gamma ',\nu ')$.

We start with defining a map $\mu :V(supp(\gamma )_{\gamma })\rightarrow \{\bullet , \circ \}$ as follows. Let $v\in V(supp(\gamma )_{\gamma })$ and let $q:=ab|cd$ be the quartet tree in $supp(\gamma )$ such that *v* is a vertex in the partially subdivided quartet tree $(q,\gamma (q))$. If *v* is a leaf of *q* then we put $\mu (v)=\circ$. So assume that *v* is not a leaf of *q*. Then *v* must be an interior vertex of *q*. We distinguish between the cases that $q\in {\mathcal {Q}}(\sigma )^-$, that $q\in {\mathcal {Q}}(\sigma )-{\mathcal {Q}}(\sigma )^-$ , and that $q\not \in {\mathcal {Q}}(\sigma )$.

If $q\in {\mathcal {Q}}(\sigma )^-$, then since $\gamma (q)=0$ both *v* and its adjacent interior vertex $v'$ must have degree three. In this case, we put $\mu (v) =\bullet$ if at least one of *v* and $v'$ is assigned the value $\bullet$ under $\nu$.

If $q\in {\mathcal {Q}}(\sigma )-{\mathcal {Q}}(\sigma )^-$ then assume first that *v* has degree three. Then *v* must be adjacent with a vertex $v'$ of degree two. If $v=med_q(a,b,c)$ then we again put $\mu (v) =\bullet$ if at least one of them is assigned the value $\bullet$ under $\nu$. Otherwise, we put $\mu (v)=\circ$. In all four cases, we also put $\mu (v')=\circ$. If *v* is of degree three but $v\not =med_q(a,b,c)$ then we put $\mu (v)=\nu (v)$. If *v* is of degree two and $\mu (v')$ has not been defined yet for this subcase then we put $\mu (v)=\nu (v)$.

If $q\not \in {\mathcal {Q}}(\sigma )$ then suppose first that *v* is a vertex of degree three. Then we may assume without loss of generality that $v=med_q(a,b,c)$. If there exists a quartet tree $q':=zb|ac\in {\mathcal {Q}}(\sigma )^-$ such that $\mu (med_{q'}(a,b,c))\not =\nu (med_{q'}(a,b,c))$ then we put $\mu (v)= \mu (med_{q'}(a,b,c))$. If *v* is not a vertex of degree three, then we put $\mu (v)=\nu (v)$. For all other cases for *v* we also put $\mu (v)=\nu (v)$.

To complete our definition of $(\gamma ',\nu ')$, we put, for all $q\in {\mathcal {Q}}(X)$, $\gamma '(q)=-1$ if $q\in {\mathcal {Q}}(\sigma )^-$ or $\gamma (q)=-1$. Otherwise, we define $\gamma '(q)$ to be the number of augmentation points of *q* under $\mu$. Clearly, $supp(\gamma ')\not =\emptyset$ because ${\mathcal {Q}}(\sigma )^-\not =supp(\gamma )$. Furthermore, since every augmentation point of a partially subdivided quartet tree in $supp(\gamma ')_{\gamma '}$ is also an augmentation point of a partially subdivided quartet tree in $supp(\gamma )_{\gamma }$, we have $supp(\gamma ')_{\gamma '}\subseteq supp(\gamma )_{\gamma }$. In view of this, we define $\nu ':V(supp(\gamma ')_{\gamma '}) \rightarrow \{\bullet ,\circ \}$ as the restriction $\mu |_{V( supp(\gamma ')_{\gamma '})}$ of $\mu$ to $V(supp(\gamma ')_{\gamma })$.

Note that $(\gamma ',\nu ')$ is also an enhanced quartet tree system on *X* because $|supp(\gamma )|\ge 2$, and that $supp(\gamma ')_{\gamma '}\subseteq supp(\gamma )_{\gamma }$ also holds for $(\gamma ',\nu ')$. To help keep notation at bay, we will from now on only mean $\nu '$ when referring to an augmentation point/vertex of a quartet tree in $supp(\gamma ')_{\gamma '}$. Note also that $q^*\not \in supp(\gamma ')$ because $q^*\in {\mathcal {Q}}(\sigma )^-$. Hence, $|supp(\gamma ')|<|supp(\gamma )|$.

We illustrate the definitions of the maps $\mu$, $\gamma '$ and $\nu '$ by means of the enhanced quartet tree system $(\gamma ,\nu )$ on $X:=\{a,b,c,d,e\}$ in the left column of the table in Figure 5(i). A routine to check shows that $(\gamma ,\nu )$ satisfies Properties (A1) - (A6). Using the notation introduced for this direction of the proof, we take the quartet trees *p* and $q^*$ to be $q_4$ and the split $\sigma$ to be *abc*|*de*. Then ${\mathcal {Q}}(\sigma )^-= \{q_4, q_5\}$. The map $\mu$ is given in the middle column of the table in Figure 5(i) and the enhanced quartet tree system $(\gamma ',\nu ')$ is presented in the right column of that table. Since $(\gamma ',\nu ')$ satisfies Properties (A1)-(A6) it follows that there exists an augmented tree ${\mathcal {T}}'$ on *X* with underlying tree $T'$ such that $\gamma _{T'}=\gamma '$ and $\nu _{T'}=\nu '$. That augmented tree is depicted at the top of Figure 5(ii). By replacing the vertex *w* with an edge $e=\{w',w''\}$ so that the split $\sigma$ is also displayed by $T'$ and assigning the values $\bullet$ and $\circ$ to the vertices incident with *e* based on the values of the interior vertices of a quartet tree in ${\mathcal {Q}}(\sigma )^-$ results in the augmented tree ${\mathcal {T}}$ at the bottom of Figure 5(ii). With denoting the underlying tree of ${\mathcal {T}}$ by *T*, we obtain $\gamma _T=\gamma$ and $\nu _T=\nu$.

Clearly, $(\gamma ', \nu ')$ satisfies Property (A1) because $(\gamma , \nu )$ satisfies (A1). To see that $(\gamma ',\nu ')$ satisfies Property (A2), assume for contradiction that there exist elements $a,b,c,d\in X$ with $ab|cd\in supp(\gamma ')$ but (A2) does not hold. Then, for some $x\in X-\{a,b,c,d\}$, $i\in \{c,d\}$, and $j\in \{a,b\}$, we have

6 $$\begin{aligned} ab|ix, jx|cd\not \in supp(\gamma '). \end{aligned}$$

Without loss of generality, we may assume that $i=c$ and that $j=b$. Then $ab|cx, bx|cd\not \in supp(\gamma ')$. Since $ab|cd\in supp(\gamma ')\subseteq supp(\gamma )$ and $(\gamma ,\nu )$ satisfies (A2),

$$ ab|cx\in supp(\gamma ) \text{ and } ab|dx \in supp(\gamma ) $$

or

$$ ax|cd\in supp(\gamma ) \text{ and } bx|cd\in supp(\gamma ) $$

must hold. The definitions of $\gamma '$ and $\nu '$ combined with Assumption (6) imply that *ab*|*cx* and *bx*|*cd* are both contained in $Q(\sigma )^-$. But $ab|cx\in Q(\sigma )^-$ implies that *x* and *c* are contained in the same part of $\sigma$ and $bx|cd \in Q(\sigma )^-$ implies that *x* and *c* are not contained in the same part of $\sigma$; a contradiction. Thus, $(\gamma ',\nu ')$ satisfies (A2).

To see that $$(\gamma ',\nu ')$$ satisfies Property (A3), let $$q,q'\in supp(\gamma ')$$ be such that there exist three pairwise distinct leaves $$x,y,z\in L(q)\cap L(q')$$. Put $$v=med_q(x,y,z)$$ and $$w=med_{q'}(x,y,z)$$ and let *a* and $$a' $$ denote the remaining leaf of *q* and $$q'$$, respectively. Then $$\nu (v)=\nu (w)$$ because $$(\gamma ,\nu )$$ satisfies (A3). Assume for contradiction that $$\nu '(v)\not =\nu '(w)$$. Then for one of *v* and *w*, say *v*, we have $$\nu '(v)=\bullet $$ and $$\nu '(w)=\nu (w)=\nu (v)=\circ $$. To complete the proof that $$(\gamma ',\nu ')$$ satisfies Property (A3), we distinguish between the cases that $$q\in {\mathcal {Q}}(\sigma )$$ and that $$q\in {\mathcal {Q}}(X)-{\mathcal {Q}}(\sigma )$$.

Assume first that $q\in {\mathcal {Q}}(\sigma )$. Then $q\in {\mathcal {Q}}(\sigma )-{\mathcal {Q}}(\sigma )^-$ because $q\in supp(\gamma ')$. Then since $(\nu ,\gamma )$ satisfies Property (A4), every augmentation point of the partially subdivided quartet tree $(q,\gamma (q))$ under $\nu$ is supported by $supp(\gamma )$. Combined with the definition of the map $\mu$ and the fact that $\nu (v)=\circ$ and $\nu '(v)=\bullet$, it follows that we may assume without loss of generality that there exists a vertex $u'$ in $(q,\gamma (q))$ that is adjacent with *v* and an element $b\in X-\{x,y,z,a,a'\}$ such that the quartet tree $q'':=xb|yz$ is contained in ${\mathcal {Q}}(\sigma )^-$. Note that $u'$ is either an augmentation vertex or an augmentation point of $(q,\gamma (q))$ under $\nu$.

**Fig. 6:**
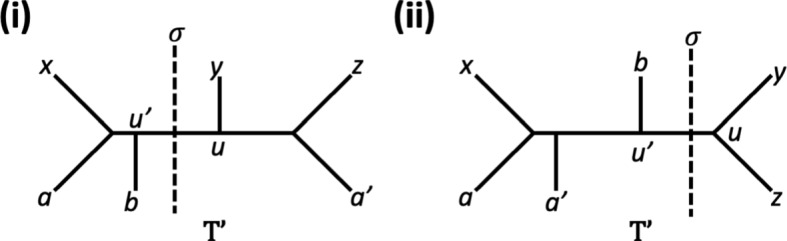
The phylogenetic trees $T'$ on $\{x,y,z,a,a',b\}$ considered in the proof part of Theorem 1 concerned with showing that the enhanced quartet tree system $(\gamma ',\nu ')$ satisfies Property (A3) for the case that the quartet tree *q* is contained in ${\mathcal {Q}}(\sigma )-{\mathcal {Q}}(\sigma )^-$. (i) The case that *a* and $a'$ are such that $q=xa|yz$ and $q'=xy|za'$. (ii) The case that *a* and $a'$ are such that $q=xa|yz$ and $q'=xa'|yz$. In both cases, the split $\sigma$ is indicated by a dashed line and the vertex *u* results in *v* and *w* when restricting $T'$ to *L*(*q*) and $L(q')$, respectively

If *a* and $a'$ are such that $q=xa|yz$ and $q'=xy|za'$ then the phylogenetic tree on $Y:=\{x,y,z,a,a',b\}$ obtained from $T(q,q')$ by adding $u'$ and *b* as indicated in Figure 6(i) is the sole phylogenetic tree on *Y* that simultaneously displays *q*, $q'$ and $q''$. Thus, $q'\not \in {\mathcal {Q}}(\sigma )^-$. Since $\mu (med_{q''}(x,y,z))=\bullet \not =\circ =\nu (med_{q''}(x,y,z))$ it follows by the definition of $\mu$ that $\circ =\mu (w)=\mu (med_{q''}(x,y,z))=\bullet$ which is impossible.

If *a* and $a'$ are such that $q=xa|yz$ and $q'=xa'|yz$ then, assumig without loss of generality that $\lambda (q)\ge \lambda (q')$, it follows that the phylogenetic tree on $Y:=\{x,y,z,a,a',b\}$ obtained from $T(q,q')$ by adding $u'$ and *b* as indicated in Figure 6(ii) is the sole phylogenetic tree on *Y* that simultaneously displays *q*, $q'$ and $q''$. Note that $a'=b$ might hold. Hence, $u'$ must also be an augmatation point or augmentation vertex of the partially subdivided quartet tree $(q',\gamma (q'))$ under $\nu$. By the definiton of $\mu$ it follows that $\bullet =\nu '(v)=\mu (v)=\mu (w)=\nu '(w)=\circ$ which is impossible.

Assume for the remainder of this proof part of the theorem that $q\in {\mathcal {Q}}(X)-{\mathcal {Q}}(\sigma )$. Then since $\nu (v)=\circ =\nu (w)$ and $\nu '(v)=\bullet$ it follows that there must exist a subdivision vertex $u'$ in the partially subdivided quaret tree $(q',\gamma (q'))$ that is an augmentation vertex under $\nu$ and that is adjacent with *w*. Since $u'$ is supported by $supp(\gamma )$ because $(\gamma ,\nu )$ satisfies Property (A4), it follows that there exists an element $b\in X-Y$ where *Y* is defined as in the previous case such that the quartet tree *xy*|*bz* is contained in ${\mathcal {Q}}(\sigma )^-$. By the definition of $\mu$ it follows that $\circ =\nu '(w)=\mu (w)=\bullet$ which is impossible. This completes the proof that $(\gamma ',\nu ')$ satisfies (A3).

To see that $(\gamma ',\nu ')$ satisfies Property (A4), suppose there exists some quartet tree $q=ab|cd\in supp(\gamma ')$ such that $\gamma '(q)\ge 1$. Let *s* be an augmentation vertex of the partially subdivided quartet tree $(q,\gamma (q))$ under $\nu '$. Since $supp(\gamma ')_{\gamma '}\subseteq supp(\gamma )_{\gamma }$, Property (A4) applied to $(\gamma ,\nu )$ implies that *s* is also an augmentation point of $(q,\gamma (q))$ under $\nu$ and that *s* is supported in $supp(\gamma )$. Hence, there exist quartet trees $q':=bz|cd$ and $q'':= ab|dz$ in $supp(\gamma )$ such that *q* is displayed by $T=T(q',q'')$, the median $med_T(b,z,d)$ in *T* induces the median $k'=med_{q'}(b,d,z)$ in $q'$ when restricting *T* to $\{b,c,d,z\}$, $k''=med_{q''}(b,d,z)$ is the median in $q''$ when restricting *T* to $\{a,b,d,z\}$, and $\nu (k') =\nu (k'') =\bullet$. Since at most one of $q'$ and $q''$ can be contained in ${\mathcal {Q}}(\sigma )^-$, it follows that to establish that $(\gamma ',\nu ')$ satisfies Property (A4), we only need to consider the cases that $q'$ and $q''$ are both also contained in $supp(\gamma ')$ and that one of them is not contained in $supp(\gamma ')$.

If $q'$ and $q''$ are also contained in $supp(\gamma ')$ then neither $q'$ nor $q''$ is a quartet tree in ${\mathcal {Q}}(\sigma )^-$. Hence, $\nu '(k')=\nu (k')=\bullet =\nu (k'')=\nu '(k'')$ and so (A4) holds for $(\gamma ',\nu ')$ in this case. So assume that one of $q'$ and $q''$ is not contained in $supp(\gamma ')$ and so is a quartet tree in ${\mathcal {Q}}(\sigma )^-$. Without loss of generality, assume that $q'\in {\mathcal {Q}}(\sigma )^-$. Then $\nu '(k')=\bullet$ by the definition of $\nu '$, and $q''\not \in {\mathcal {Q}}(\sigma )$. Hence, $q''\in supp(\gamma ')$ and so $\nu '(k'') =\nu (k) =\bullet$ must also hold which, in turn, implies $\nu '(k')=\bullet =\nu '(k'')$. Thus, $(\gamma ',\nu ')$ satisfies (A4) also in this case.

**Fig. 7:**
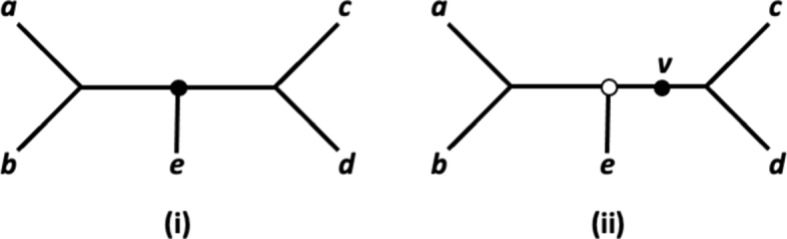
The two generic versions of the augmented partially subdivided tree $\overline{{\mathcal {T}}}=(\overline{T}, \overline{\nu })$ on $Y=\{a,b,c,d,e\}$ mentioned in the proof that $(\gamma ',\nu ')$ satisfies Property (A5). In both cases, augmentations of the leaves and potential further augmentation points are omitted

To see that $(\gamma ',\nu ')$ satisfies Property (A5), suppose that $a,b,c,d,e \in X$ are such that *ab*|*cd* and $q:=ab|ce$ are quartet trees that are both contained in $supp(\gamma ')$ and that $0\le \gamma '(q)<\gamma '(ab|cd)$. Then any augmented partially subdivided tree $\overline{{\mathcal {T}}}=(\overline{T}, \overline{\nu })$ on $Y=\{a,b,c,d,e\}$ such that $\gamma _{\overline{T }}=\gamma '|_Y$ and $\nu _{\overline{T }}=\nu '|_Y$ must be of the form indicated in Figure 7(i) and (ii) where, in both cases, we have omitted the augmentations of the leaves and also potential further augmentation points.

We first claim that $0\le \gamma (q)<\gamma (ab|cd)$. Since $0 \le \gamma '(q)\le \gamma (q)$ clearly holds, it suffices to show that $\gamma (q)<\gamma (ab|cd)$ holds. Assume for contradiction that $\gamma (ab|cd)\le \gamma (q)$. Then since, for any quartet tree $p\in supp(\gamma ')$, the definition of $\gamma '$ implies that the difference $\gamma (p)-\gamma '(p)$ is zero or one, a routine check yields $\gamma '(q)=\gamma (q)-1$ and $\gamma '(ab|cd)=\gamma (ab|cd)$. Hence, $q\in {\mathcal {Q}}(\sigma )$. Indeed, let $v_1,\ldots , v_{\gamma (q)}$ denote the augmentation points of the partially subdivided quartet tree $(q,\gamma (q))$ under $\nu$ where $v_1$ is adjacent with $med_q(a,b,c)$. Since each augmentation point of $(q,\gamma (q))$ is supported by $supp(\gamma )$ by Property (A4), there exist quartet trees $q_{v_1}$ and $q_{v_1}'$ in $supp(\gamma )$ that support $v_1$. Without loss of generality, we may assume that $a,b\in L(q_{v_1})$. Since $v_2,\ldots , v_{\gamma (q)}$ are also augmentation points of the partially subdivided quartet tree $(q_{v_1}',\gamma (q_{v_1}'))$ under $\nu$, we can repeat this process of finding support quartet trees for augmentation points of $(q,\gamma (q))$ with $(q,\gamma (q))$ replaced by $(q_{v_1}',\gamma (q_{v_1}'))$ and $v_1$ replaced by $v_2$ to obtain a sequence $\omega '$ of augmented quartet trees under $\nu$ none of which contains an augmentation point of $(q,\gamma (q))$. Since $\gamma '(q) =\gamma (q)-1$, the definition of $\gamma '$ implies that $\omega '$ must contain a quartet tree that is contained in ${\mathcal {Q}}(\sigma )^-$. Hence, $q\in {\mathcal {Q}}(\sigma )$, as required.

In view of Figure 7, it follows that the analogously defined sequence of augmented quartet trees under $\nu$ for *ab*|*cd* must also contain a quartet tree in ${\mathcal {Q}}(\sigma )^-$. Thus, $\gamma (ab|cd)-1=\gamma '(ab|cd)=\gamma (ab|cd)$ which is impossible. This completes the proof of the claim.

To complete the proof that $(\gamma ',\nu ')$ satisfies Property (A5), assume first that we have $\nu '(med_q(a,c,e))=\circ$. Then $\nu (med_q(a,c,e))=\circ$ by the definition of $\nu '$. Since $(\gamma ,\nu )$ satisfies Property (A5), we obtain $\gamma (ae|cd)= \gamma (ab|cd)-\gamma ( q)$. Since *ab*|*cd* is displayed by $\sigma$ if and only if precisely one of *q* and *ae*|*cd* is displayed by $\sigma$ it follows that $\gamma '(ab|cd)=\gamma (ab|cd)-1$ if and only if $\gamma '(p)=\gamma (p)-1$ for a single quartet tree $p\in \{q,ae|cd\}$ and that $\gamma '(p')=\gamma (p')$ for the quartet tree $p'\in \{q,ae|cd\}-\{p\}$. Thus, $\gamma '(ae|cd)=\gamma '(ab|cd)-\gamma '( q)$ as required.

So assume that $\nu '(med_q(a,c,e))=\bullet$. Then, by the definition of $\nu '$, either $\nu (med_q(a,c,e))=\bullet$ or $\nu (med_q(a,c,e))=\circ$. If $\nu (med_q(a,c,e))=\bullet$ then similar arguments as in the previous case imply that $\gamma '(ae|cd)= \gamma '(ab|cd)-\gamma '( q)-1$, again as required.

So assume that $\nu (med_q(a,c,e))=\circ$. Then since $(\gamma ,\nu )$ satisfies (A5), we obtain $\gamma (ae|cd)=\gamma (ab|cd)-\gamma ( q)$. Note that $\gamma '(q)=\gamma (q)-1$. Consequently $\gamma (ae|cd)=\gamma '(ae|cd)$ because $q\in {\mathcal {Q}}(\sigma )$ and so, by (A6) applied to *q* and *ae*|*cd*, we obtain $ae|cd\not \in {\mathcal {Q}}(\sigma )$. Thus $\gamma '(ae|cd)=\gamma '(ab|cd)-\gamma '( q)-1$ again, as required. This completes the proof that $(\gamma ',\nu ')$ satisfies (A5).

To see that $(\gamma ',\nu ')$ satisfies Property (A6), assume that $a,b,c,d,e\in X$ are such that $q:=ab|cd$ and $q':=bc|de$ are quartet trees in $supp(\gamma ')$. Then *q* and $q'$ are also contained in $supp(\gamma )$ because $0\le \gamma '(p)\le \gamma (p)$ holds for all $p\in \{q,q'\}$. Note that, independent of the value of $\nu (med_{ab|cd}(b,c,d))$, we have that *ab*|*de* is also displayed by $T(q,q')$. Note that $\sigma$ displays *ab*|*de* if and only if $\sigma$ displays one of *q* and $q'$.

Suppose first that $\sigma$ does not display *ab*|*de*. Then $\gamma '(p)=\gamma (p)$, for all $p\in \{ab|de, q,q'\}$. Furthermore, $\nu (med_{ab|cd}(b,c,d))= \nu '(med_{ab|cd}(b,c,d))$. Since $(\gamma ,\nu )$ satisfies Property (A6), if follows that $(\gamma ',\nu ')$ satisfies Property (A6).

So assume that $\sigma$ displays *ab*|*de*. Without loss of generality, assume that $\sigma$ displays *q*. Then $\gamma '(q')=\gamma (q')$. If $\nu '(med_q(a,c,d))= \circ$ then $\nu (med_q(a,c,d))= \circ$ and $\gamma (p)=\gamma '(p)$, for all $p\in \{ab|de,q\}$. Hence, $(\gamma ',\nu ')$ satisfies Property (A6) because $(\gamma ,\nu )$ satisfies that property.

So assume that $\nu '(med_q(a,c,d))= \bullet$. Then, by the definition of $\nu '$, either $\nu (med_q(a,c,d))= \bullet$ or $\nu (med_q(a,c,d))= \circ$. In the former case, $\gamma (ab|de)=\gamma '(ab|de)+1$ and $\gamma '(q)=\gamma (q)-1$. Similar arguments as before imply that $(\gamma ',\nu ')$ satisfies Property (A6) also in this case. Finally, if $\nu (med_q(a,c,d))= \circ$, then $\gamma (ab|de)=\gamma '(ab|de)$ and $\gamma '(q)=\gamma (q)-1$. Since $(\gamma ,\nu )$ satisfies Property (A6), we obtain $\gamma '(ab|de)=\gamma (ab|de)=\gamma (q)+\gamma (q')=\gamma '(q) +\gamma '(q')+1$. Hence, $(\gamma ',\nu ')$ satisfies Property (A6) also in this case.

In summary, we have that $(\gamma ',\nu ')$ is an enhanced quartet tree system that satisfies Properties (A1) - (A6).

Since $|supp(\gamma ') | < |supp(\gamma ) |$ and $(\gamma ',\nu ')$ satisfies (A1)-(A6) it follows by induction hypothesis that there exists an augmented tree ${\mathcal {T}}'$ on *X* with underlying tree $T'$ such that $\gamma _{T'}=\gamma '$ and $\nu _{T'}=\nu '$. By the Splits-Equivalence Theorem reviewed above, it follows that $\varSigma (T')$ is compatible. Note that, by construction, $\sigma \not \in \varSigma (T')$. But then $\varSigma (T')\cup \{\sigma \}$ must be compatible as otherwise the close relationship between quartet trees and splits briefly reviewed in Section 2.2 implies that there exist four pairwise distinct elements *x*, *y*, *z*, and *r* in *X* such that one of the three possible quartets trees with leaf set $\{x,y,z,r\}$ is contained in $supp(\gamma ')$ and another is contained in ${\mathcal {Q}}(\sigma )^-$. But this is impossible because both quartet tree systems are contained in $supp(\gamma )$ and $(\gamma ,\nu )$ satisfies (A1). By the Splits-Equivalence Theorem, it follows that there exists a phylogenetic tree *T* on *X* such that the quartet tree system ${\mathcal {Q}}(T)$ induced by *T* equals $supp(\gamma )$.

Let $e:=\{w',w''\}$ denote the edge in *T* whose deletion induces the split $\sigma$ on *X*. Since $T'$ is clearly obtained from *T* by collapsing *e*, we obtain $V(T)=V(T')-\{med_{T'}(s,s',t)\} \cup \{w',w''\}$. Since $med_T(s,s',t)$ and $med_T(s,t,t')$ are both incident with *e*, we may assume without loss of generality that $w'=med_T(s,s',t)$ and that $w''=med_T(s,t,t')$. Consider the augmentation map $\hat{\nu }:V(T)\rightarrow \{\bullet , \circ \}$ given, for all $v\in V(T)$, by putting $\hat{\nu }(v)=\nu _{T'}(v)$ if $v\in V(T')- \{med_{T'}(s,s',t)\}$ and $\hat{\nu }(v)= \nu (med_{q^*}(s,s',t))$ if $v=w'$ and $\hat{\nu }(v)= \nu (med_{q^*}(t,t',s))$ if $v=w''$. Since $\hat{\nu }$ induces $\nu _T$ and $\nu '=\nu _{T'}$ and $\gamma '=\gamma _{T'}$ hold, it follows by the definition of $\nu '$ that $\nu =\nu _T$ and $\gamma =\gamma _T$, as required.

The remainder of the theorem is a straightforward consequence of the fact that any two phylogenetic trees *T* and $T'$ such that ${\mathcal {Q}}(T)$ equals ${\mathcal {Q}}(T')$ must be equivalent, where by equal we mean that for every quartet tree *q* in ${\mathcal {Q}}(T)$ there must exist a (necessarily unique) quartet tree in ${\mathcal {Q}}(T')$ that is equivalent with *q* and vice versa (see (Semple et al. (2003), Corollary 6.3.8)) and the fact that for any augmented tree ${\mathcal {T}}$ with underlying tree *T* we must have that $\nu _T=\nu$ and $\gamma _T =\gamma$. $\square$

## Augmented trees and arboreal networks

We now turn our attention to arboreal networks which we already described briefly in the introduction. Formally speaking, an *arboreal network* *N* (*on* *X*) is a multiple-rooted directed acyclic graph with leaf set *X* such that every vertex with indegree at least two has outdegree one and planting *N* and ignoring directions results in a phylogenetic tree on $X\cup R(N)$. In this context, *R*(*N*) denotes the set of roots $\widehat{r}$ of the planted version of *N* that were attached via incoming arcs $(\widehat{r},r)$ to the roots *r* of *N*. Note that, by construction, $R(N)\not =\emptyset$. Clearly, every planted arboreal network *N* can be transformed back into the arboreal network that gave rise to it by reversing the planting process described in Section 1, that is, removing for every root *r* of *N* the newly added vertex $\widehat{r}$ and the arc $(\widehat{r},r)$. Although, for every arboreal network *N* its planted version $N^p$ gives rise to an augmented tree $\mathcal {U}(N):= \mathcal {U}(N^p)$ as described in the introduction, not every augmented tree gives rise to a planted arboreal network and, therefore, to an arboreal network. For example, since an augmentation vertex in $\mathcal {U}(N)$ is a reticulation vertex in *N* and a reticulation vertex in an arboreal network has outdegree one and neither one of its parents can be a root of the network, no augmentation vertex in $\mathcal {U}(N)$ can be adjacent with more than one leaf.

**Fig. 8:**
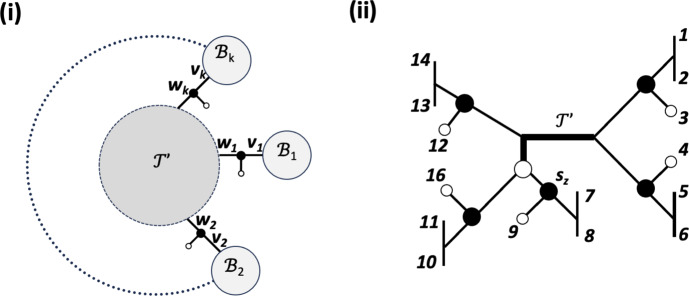
(i) A configuration for a binary augmented tree ${\mathcal {T}}$ on *X* without adjacent augmentation vertices that prevents ${\mathcal {T}}$ from being transformable into an arboreal network – see text for details. (ii) For $k=5$, an example of an augmented tree on $X:=\{1,\ldots , 14, 16\}$ in the form of the forbidden configuration in (i). For all $1\le i\le 5$, the disks $\mathcal {B}_i$ are multi-cherries of size two, $\mathcal {T}'$ is the path indicated in bold, and the vertices $w_i$ are indicated as $\bullet$

Even though this insight is undoubtedly useful, from the point of view of reconstructing an arboreal network *N* from an augmented tree ${\mathcal {T}}=(T,\nu )$ such that ${\mathcal {T}}$ is augmentation-equivalent with the augmented tree $\mathcal {U}(N)$ associated to *N* it is however not sufficient, even if ${\mathcal {T}}$ is binary and does not contain augmentation vertices that are adjacent with each other. One of the reasons for this is the configuration for ${\mathcal {T}}$ depicted in Figure 8(i) where the vertices indicated by "$\circ$" are leaves of ${\mathcal {T}}$ and, for some $k\ge 3$, the grey disk marked ${\mathcal {T}}'$ is an augmented tree on the set of vertices of ${\mathcal {T}}$ that are adjacent with one of the augmentation vertices $w_i$ of ${\mathcal {T}}$ but are not contained in $\mathcal {B}_i$, $3\le i\le k$, or a leaf. For all $3\le i\le k$, some integer $k\ge 3$, the disk marked $\mathcal {B}_i$ is the augmented tree obtained by (i) attaching a new leaf $l_{v_i}$ to the vertex $v_i$ in the underlying tree of $\mathcal {B}_i$ that is adjacent with $w_i$. (ii) assigning the value $\circ$ to $l_{v_i}$ and preserving all other values under $\nu$, and (iii) deleting the edge $\{v_i,w_i\}$ from *T*.

More precisely, assume that ${\mathcal {T}}$ is a binary augmented trees such that no two augmentation vertices are adjacent and that is of the form as described in that figure. Then since ${\mathcal {T}}$ is binary and a reticulation vertex in an arboreal network has oudegree one, it is straightforward to see that, once oriented, ${\mathcal {T}}'$ must contain a vertex that has outdegree three. Indeed, continuing with the notation in Figure 8(i), each edge incident with a vertex in ${\mathcal {T}}'$ and a vertex $w_i$, $1\le i\le k$, some $k\ge 3$ must be directed towards $w_i$ because $w_i$ is an augmentation vertex of ${\mathcal {T}}$ that is adjacent with a leaf. Since $k\ge 3$ and ${\mathcal {T}}'$ does not contain a leaf and therefore no leaf that could serve as a root in an arboreal network it follows that, once oriented, ${\mathcal {T}}'$ has a vertex of outdegree three, as required. Thus, there does not exist an arboreal network *N* such that $\mathcal {U}(N)$ is augmentation-equivalent with ${\mathcal {T}}$.

**Fig. 9:**
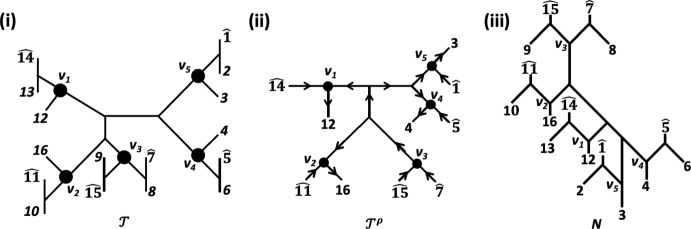
(i) An augmented tree ${\mathcal {T}}=(T,\nu )$ on $X\cup R$ where *R* comprises the leaves of the form ${\widehat{i}}$, $i\in \{1, 5,7,11,14,15\}$ and *X* is the set of remaining leaves. As before, the augmentation vertices of ${\mathcal {T}}$ under $\nu$ are indicated by "$\bullet$" and the value of all other vertices under $\nu$ is omitted. (ii) Ignoring the direction of the edges, the augmented tree ${\mathcal {T}}^{\rho }$ for $\rho =\widehat{1}$. (iii). An arboreal network *N* obtained by algorithm Check when given *X*, *R*, and the augmented tree ${\mathcal {T}}$ on $X\cup R$ as indicated in (i)

On the other hand, if ${\mathcal {T}}$ is such that one of the leaves in the configuration in Figure 8(i) is an augmented tree with at least two vertices all of which are non-augmentation vertices then it is not difficult to to see that there exists an arboreal network *N* such that $\mathcal {U}(N)$ and ${\mathcal {T}}$ are augmentation equivalent because this extra leaf introduces a level of flexibility for directing edges. To help illustrate this point, consider the augmented tree in Figure 9(i) which is obtained from the augmented tree ${\mathcal {T}}$ in Figure 8(ii) by replacing the leaf "9" with the multi-cherry $\{9,\widehat{15}\}$. Then it is not difficult to check that ${\mathcal {T}}$ is augmentation-equivalent with the augmented tree $\mathcal {U}(N)$ obtained from the arboreal network *N* in Figure 9(iii). However even with this requirement for two leaves there does not exist, in general, an arboreal network *N* such that $\mathcal {U}(N)$ and ${\mathcal {T}}$ are augmentation-equivalent if ${\mathcal {T}}'$ contains at least one augmentation vertex.

We conclude this section with presenting an efficient algorithm called Check which, in case the set of roots of an arboreal networks (or more precisely the set *R* of roots of its planted version) is known, will construct an arboreal network *N* with leaf set *X* from an augmented tree ${\mathcal {T}}{:=(T,\nu )}$ on $X\cup R$ such that *R* is the root set of $N^p$ and ${\mathcal {T}}$ and $\mathcal {U}(N)$ are augmentation-equivalent. To this end, we construct an augmented tree ${\mathcal {T}}^\rho {:=(T^{\rho }, \nu ^{\rho }:=\nu |_{ V(T^{\rho })} )}$ on $R\cup L_{\mathcal {A}_{{\mathcal {T}}}}$ as follows were $L_{\mathcal {A}_{{\mathcal {T}}}}$ denotes the set of leaves of $T^{\rho }$ that are adjacent with an augmentation vertex of $T^{\rho }$. We first make a copy of ${\mathcal {T}}$ which we call ${\mathcal {T}}_c$. We then choose an element $\rho \in R$ and, starting at $\rho$, use a depth-first search to traverse ${\mathcal {T}}_c$ to make the following modifications to the underlying tree $T_c$ of ${\mathcal {T}}_c$ and *T* which is based on the fact that interior vertices of $T_c$ are visited at least twice by our search. For each vertex *w* of $T_c$ not in $\mathcal {A}_{{\mathcal {T}}}\cup R$ such that between visits *i* and $i+1$ of *w*, some $i\ge 1$, only vertices not in $\mathcal {A}_{{\mathcal {T}}}\cup R$ are encountered, we remove all encountered vertices (and their incident edges!) from $T_c$ and suppress *w* in $T_c$. For each such *w*, we mirror this modification of $T_c$ in *T* by directing all removed edges towards the removed leaves. The augmented tree that results once the traversal of ${\mathcal {T}}_c$ is complete is ${\mathcal {T}}^\rho$. For example, for the augmented tree ${\mathcal {T}}$ in Figure 9(i) and $R=\{\widehat{1}, \widehat{5}, \widehat{7}, \widehat{15},\widehat{11}, \widehat{14}\}$, ignoring the orientations of the edges for the moment, the augmented tree ${\mathcal {T}}^\rho$ on $R \cup L_{\mathcal {A}_{{\mathcal {T}}}}$ is depicted in Figure 9(ii) where $\mathcal {A}_{{\mathcal {T}}}=\{v_1,\ldots v_5\}$ and $L_{\mathcal {A}_{{\mathcal {T}}}}=\{3,4,12, 16\}$.

Continuing with this notation, we next state our algorithm Check where a *2-split* of *X* is a split *A*|*B* of *X* such that $\min \{|A|,|B|\}=2$. To help improve its exposition, we associate a look-up table $\tau _{{\mathcal {T}}}$ to ${\mathcal {T}}$ during the construction of ${\mathcal {T}}^{\rho }$ that records which edges $e:=\{u,v\}$ in $T^{\rho }$ correspond to paths $P_e$ in *T* of length at least two joining *u* and *v*.

**Algorithm 1:**
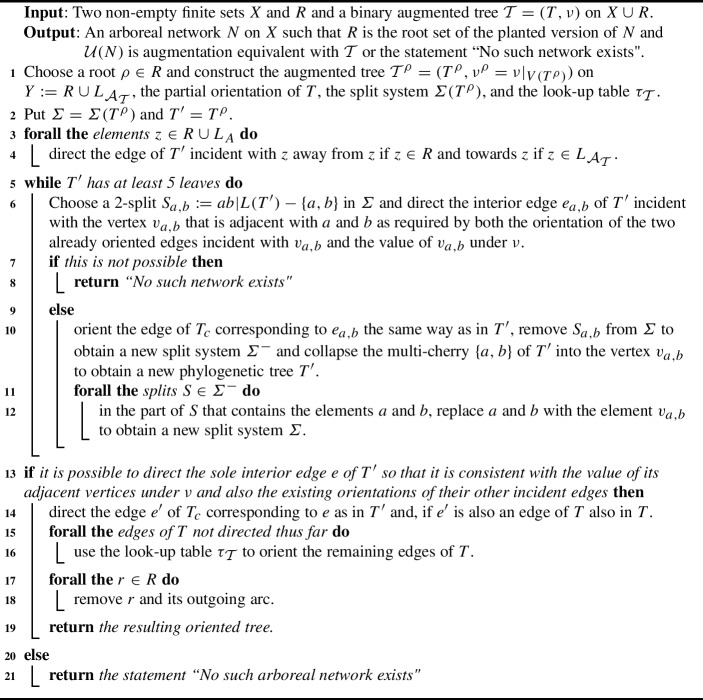
Check

We illustrate the algorithm Check by returning to the augmented tree ${\mathcal {T}}$ in Figure 9(i). For *R* and *X* as in the caption of that figure and putting $\rho = \widehat{1}$, the augmented tree on $Y:= R\cup L_{\mathcal {A}_{{\mathcal {T}}}}$ depicted in Figure 9(ii) with the orientation of the edges ignored is ${\mathcal {T}}^{\rho }$. The split system $\varSigma (T^{\rho })$ comprises all trivial splits on *Y* as well as the 2-splits $S_{\widehat{1},3}$, $S_{\widehat{5},4}$, $S_{\widehat{7},\widehat{15}}$, $S_{\widehat{11},16}$, and $S_{\widehat{14},3}$, and the splits $S_{\widehat{11},16,\widehat{15},\widehat{7}}$ and $S_{\widehat{1},3,\widehat{5},4}$ (Line 1), where for a non-empty proper subset $A:=\{a_1,\ldots , a_k\}$ of some set *Z*, some $k\ge 1$, we denote the split $a_1a_2\ldots a_k|Z-A$ by $S_{a_1,\ldots , a_k}$. Then $T'=T^{\rho }$ and $\varSigma =\varSigma (T^{\rho })$ (Line 2).

The loop starting in Line 3 directs all edges of $T'$ incident with a leaf in *Y* as described in Line 4 thus turning $T'$ into a partially directed phylogenetic tree on *Y*. In the first pass through the while loop starting at Line 5, we choose the 2-split $S_{\widehat{1},3}$ and direct the edge $e_{\widehat{1},3}$ of $T'$ as indicated in Figure 9(ii) because $v_{\widehat{1},3}$ is the augmentation vertex $v_5$ of ${\mathcal {T}}^{\rho }$ and so must have a further incoming arc as it already has an outgoing arc. We then orient the edge of *T* corresponding to $e_{\widehat{1},3}$ in the same way as in $T'$, put $\varSigma ^-=\varSigma -\{S_{\widehat{1},3}\}$ and collapse the multi-cherry $\{\widehat{1},3\}$ into $v_5$ to obtain a (partially directed) augmented tree $T'$ on $Y-\{\widehat{1},3\}\cup \{v_5\}$ (Line 10). Denoting the leaf set also by *Y*, we update the split system $\varSigma$ as described in Line 11, i.e. apart from $S_{\widehat{1},3}$ and the original trivial splits $\widehat{1}|Y-\{\widehat{1}\}$ and $3|Y-\{3\}$, $\varSigma$ now comprises the split $v_5|Y-\{v_5\}$ and all other splits of the original split system $\varSigma$ modified as described in Line 12. Since $|L(T')|\ge 5$, we return to Line 5 and choose the thus modified original 2-split $S_{\widehat{5},4}$ which is also a 2-split for the new phylogenetic tree $T'$ and proceed as before. Processing the modified original 2-splits of $T^{\rho }$ results in the (partially directed) phylogenetic tree $T'$ with leaf set $\{v_1,v_2,v_3,v_4,v_5\}$ and multi-cherries $\{v_2,v_3\}$ and $\{v_4,v_5\}$ for which the edge incident with $v_3$ is directed away from $v_3$ and all other edges incident with a leaf are directed towards that leaf. Since $|L(T')|\ge 5$, we enter the while loop in Line 5 again and choose the 2-split $S_{v_2,v_3}$. Since the vertex $v_{v_2,v_3}$ of $T'$ is not an augmentation vertex in ${\mathcal {T}}^{\rho }$, the edge $e_{v_2,v_3}$ of $T'$ is directed away from $v_{v_2,v_3}$ (Line 6). We then direct the edge of *T* corresponding to $e_{v_2,v_3}$ in the same way as in $T'$, remove the split $S_{v_2,v_3}$ from the current split system $\varSigma$ (which is $\varSigma (T')$) to obtain $\varSigma ^-$ and then collapse the multi-cherry $\{v_2,v_3\}$ into the vertex $v_{v_2,v_3}$ to obtain a new (partially directed) phylogenetic tree $T'$ on *Y* which is now $\{v_1,v_{v_2,v_3},v_4,v_5\}$ (Line 10). We then use $\varSigma ^-$ to recompute the split system $\varSigma$ in Line 11 which now comprises all trivial splits of *Y* and the split $v_1v_{v_2,v_3}|v_4v_5$. Since $|L(T')|=4$ we do not enter while loop and orient the interior edge *e* of $T'$ towards the vertex adjacent with $v_4$ and $v_5$ as that orientation is consistent with the values of the interior vertices of $T'$ under $\nu$ and also the existing orientations of their other incident edges. We then direct the edge of *T* corresponding to *e* in the same way as in $T'$ (Line 14). Since for every leaf in $\{2, 6,8,9,10,13\}$ the edge of *T* incident with it was directed during the construction of the partial orientation of *T* (Line 1), the loop starting in Line 15 is not entered. Finally, we remove the vertices $\widehat{i}$ with $i\in \{1,5,7,11,14\}$ and their outgoing arcs from the now fully directed *T* (Lines 17 and 18) and return the resulting oriented tree (Line 19).

### Theorem 2

Suppose that *X* and *R* are two finite non-empty sets and that ${\mathcal {T}}$ is a binary augmented tree on $X\cup R$. Then algorithm Check can be used to check in $O(|X\cup R|^2)$ time if there exists an arboreal network *N* on *X* such that *R* is the root set of the planted version of *N* and $\mathcal {U}(N)$ is augmentation-equivalent with ${\mathcal {T}}$.

### Proof

By construction, algorithm Check is correct. Since the running time complexity of a depth-first-search of a tree is *O*(*m*) where *m* is the number of edges of that tree it follows that the running time complexity of constructing $${\mathcal {T}}^{\rho }$$, the partial orientation of the underlying tree of $${\mathcal {T}}$$, and the look-up table $$\tau _{{\mathcal {T}}}$$ is $$O(|X\cup R|) $$ since a binary unrooted phylogenetic tree with *m* leaves has $$2m-3$$ edges (Semple et al. (2003), Proposition 2.1.3). Furthermore, the split system $$\varSigma (T^{\rho })$$ can be computed in $$O(|Y|^2)$$ time where $$Y:=R\cup L_{\mathcal {A}_{{\mathcal {T}}}}$$ since we can choose an element $$x\in Y$$, direct all edges of $$T^{\rho }$$ away from *x*, delete all arcs, one at a time, in the resulting orientation of $$T^{\rho }$$ to obtain its associated cluster system $$\mathcal {C}$$, and then associate to each cluster $$C\in \mathcal {C}$$ the split $$C|Y-C$$. Using again the fact that a binary unrooted phylogenetic tree on *m* leaves has $$2m-3 $$ edges it follows that the loop starting in Line 11 can be carried out in *O*(|*Y*|) time and that therefore the loop starting in Line 5 can be carried out in $$O(|Y|^2)$$ time. Similarly, the loop starting in Line 15 can be carried out in *O*(|*Y*|) time since the leaf set of $$T^{\rho }$$ is *Y*. Finally, the loop starting in Line 17 can be carried out in *O*(|*R*|) time. Since $$|L_{\mathcal {A}_{{\mathcal {T}}}}|\le |X|$$ the theorem follows. $$\square $$

## Conclusion

In this paper, we have studied the question of what can be said about encodings of arboreal networks, a certain kind of multiple rooted directed acyclic graph that might prove useful for extending phylogeny-based HGT-inference methods to the case where an overall species tree might be uncertain or unavailable or the information that the transfer happened between bacteria inhabiting different ecological niches is important and therefore should be preserved. Our results are combinatorial (Theorems 1 and 2) as well as algorithmical (Algorithm 1) in nature and also include a forbidden configuration that prevents a binary augmented tree ${\mathcal {T}}$ from giving rise to an arboreal network.

Numerous questions however remain that might warrant further study. These include characterizing augmented trees that cannot be transformed, in the above sense, into an arboreal network in terms of forbidden configurations. From a more algorithmical point of view it might also be interesting to find an efficient algorithm for checking if an enhanced quartet tree system satisfies properties (A1) - (A6) and, if so, constructs an augmented tree from that gave rise to it in the sense of Theorem 1. Finally, and as a first step towards reconstructing augmented trees from real biological data and, in the long run, arboreal networks, it might be interesting to develop ways to estimate augmentation maps for quartet trees from data.

## Data Availability

Data sharing is not applicable to this article as no datasets were generated or analysed in this study.
